# Structural Basis for the Propagation of Homing Endonuclease-Associated Inteins

**DOI:** 10.3389/fmolb.2022.855511

**Published:** 2022-03-16

**Authors:** Hannes M. Beyer, Hideo Iwaï

**Affiliations:** ^1^ Institute of Biotechnology, University of Helsinki, Helsinki, Finland; ^2^ Institute of Synthetic Biology, Heinrich-Heine-University Düsseldorf, Düsseldorf, Germany

**Keywords:** intein structures, horizontal gene transfer, protein splicing, DNA recognition, intein, meganuclease, homing endonuclease

## Abstract

Inteins catalyze their removal from a host protein through protein splicing. Inteins that contain an additional site-specific endonuclease domain display genetic mobility via a process termed “homing” and thereby act as selfish DNA elements. We elucidated the crystal structures of two archaeal inteins associated with an active or inactive homing endonuclease domain. This analysis illustrated structural diversity in the accessory domains (ACDs) associated with the homing endonuclease domain. To augment homing endonucleases with highly specific DNA cleaving activity using the intein scaffold, we engineered the ACDs and characterized their homing site recognition. Protein engineering of the ACDs in the inteins illuminated a possible strategy for how inteins could avoid their extinction but spread via the acquisition of a diverse accessory domain.

## Introduction

**FIGURE 1:**
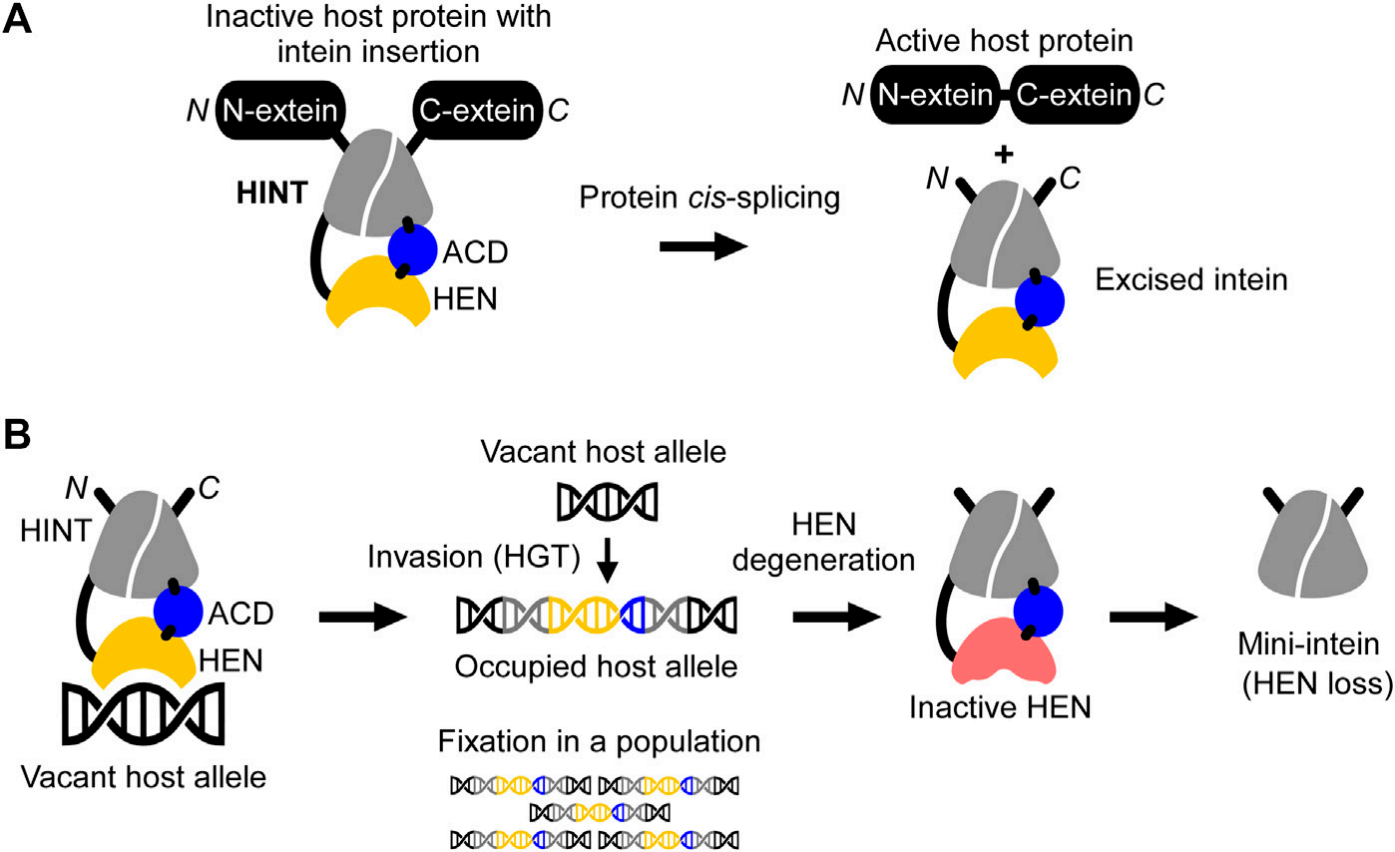
Schematic mechanisms of homing endonuclease (HEN) and Hedgehog/INTein (HINT) domains in inteins. **(A)** The HINT domain catalyzes self-excision of the intein (here consisting of HINT, HEN, and an accessory domain (ACD)) while covalently ligating the flanking extein sequences of the host protein during the protein *cis-*splicing reaction. **(B)** The nested HEN domain of the intein promotes gene conversion by introducing DNA double-strand breaks at the homing site into a vacant host allele followed by invasion via horizontal gene transfer (HGT) and fixation into the organism or population. Saturation of occupied alleles may cause HEN degeneration and loss.

Protein-splicing intervening sequences often include a homing endonuclease (HEN) domain, which is embedded within inteins containing the Hedgehog/INTein (HINT) domain (Perler, 1998). The HINT domain catalyzes the protein splicing reaction, whereas HEN domains often function independently of the HINT domain (Figure 1) (Derbyshire et al., 1997). Inteins are generally considered selfish genetic elements, frequently invading conserved coding sequences across many unicellular host organisms. In this scenario, inteins make use of homing endonuclease domains for efficient invasion by directing sequence insertion via horizontal gene transfer (HGT) initiated by DNA-strand breaks in intein-less host alleles (Figure 1B) (Barzel et al., 2011). HENs themselves are selfish genetic elements that exist free-standing (without intein or intron) or associated with inteins or introns, e.g., in group I introns (Dujon et al., 1989; Derbyshire and Belfort, 1998; Burt and Koufopanou, 2004). However, being an integral component of inteins enables HENs to invade coding sequences, which are usually more preserved than noncoding regions such as introns (Barzel et al., 2011). This association with the HINT domain becomes possible due to the unique autocatalytic protein splicing activity of inteins leading to self-removal from the host protein and ligation of the flanking protein sequences (Figure 1A). Through the association between the HINT and HEN, the latter benefits from a conserved homing environment while inteins take advantage of rapid dissemination across alleles in a given genome or population (Liu, 2000; Burt and Koufopanou, 2004; Barzel et al., 2011). Many HENs within inteins belong to the most diverse LAGLIDADG family with an extensive phylogenetic distribution (Dalgaard et al., 1997). LAGLIDADG homing endonucleases (LHEs) recognize about 14–40 bp pseudo palindromic or asymmetric target DNA sites (homing sites) and contain conserved LAGLIDADG motifs (Chevalier and Stoddard, 2001). The relatively long recognition sequence supposedly warrants high cleavage specificity, thereby reducing possible toxic effects to the host. Importantly, in contrast to most endonucleases, LHEs tolerate a certain degree of sequence variation within their homing site, an essential property for maintaining their propagation along evolutionary drifts (Argast et al., 1998).

During recent years, inteins have increasingly become important as a robust protein engineering platform thanks to their peptide-bond forming catalytic activity (Figure 1A) (Volkmann and Iwaï, 2010; Wood and Camarero, 2014). In particular, natural mini- and split inteins lacking HEN domains as well as the feasibility of splitting inteins into halves, have fostered this development (Southworth et al., 1998; Aranko et al., 2014). HEN-free mini-inteins are prevalent and have presumably emerged from HEN-associated inteins that have lost their HEN domain through size reduction (Barzel et al., 2011; Aranko et al., 2013; Novikova et al., 2016). According to the homing cycle model, HEN-less inteins may emerge after an intein has invaded and occupied all vacant alleles of a host population (“Fixation,” Figure 1B) (Burt and Koufopanou, 2004). After the fixation, HEN suffers target-site depletion and degeneration because HEN-associated inteins do not provide any benefits to host organisms, and the HEN activity is required only for invasion while protein-splicing activity is constantly selected by the production of active host proteins (Burt and Koufopanou, 2004; Barzel et al., 2011). Thus, degenerative mutations accumulate, eventually resulting in the loss of the HEN, thereby creating a mini-intein (Iwaï et al., 2017). To avoid the loss of HENs in inteins, some HEN domains might have developed a mutualism with HINT (Barzel et al., 2011; Iwaï et al., 2017). This mutualism emerged, albeit HEN and HINT were long thought as functionally independent, as seen with mini-inteins that lack HENs (Derbyshire et al., 1997). Artificially deleting HEN domains in several inteins impaired their protein splicing activity, suggesting that HEN domains, regardless of their nuclease activities, could assist in the protein splicing reaction of HINT. This domain interplay thereby might provide the selection to ensure the persistence of the HEN domain in inteins (Iwaï et al., 2017). Thus, structural and functional studies of HEN-associated inteins could shed light on the evolutionary history of individual inteins and contribute to the development of novel reagents as genomic and protein engineering tools.

In this study, we elucidated crystal structures of HEN-associated archaeal inteins inserted at the same insertion site (VMA-b), which is located within the A subunit P-loop of the vascular-type ATP synthase (VMA) from *Thermococcus litoralis* (*Tli*) and *Pyrococcus horikoshii* (*Pho*). The two three-dimensional structures highlighted a modular architecture consisting of HINT, HEN, and an accessory domain (ACD). The structures of the ACDs are diverse, even among the known three-dimensional structures of HEN-containing inteins. We further investigated the structural role of the ACD in DNA recognition of inteins by engineering the ACDs. These results suggest that the ACDs modulate DNA cleavages by the HEN-associated inteins. We speculate that acquiring a diverse ACD in HEN-associated inteins could be a general strategy to avoid their eventual extinction by promoting further spread into more distant insertion sites.

## Results

### Crystal Structures of *P. horikoshii VMA* and *T. litoralis VMA* Inteins

**FIGURE 2:**
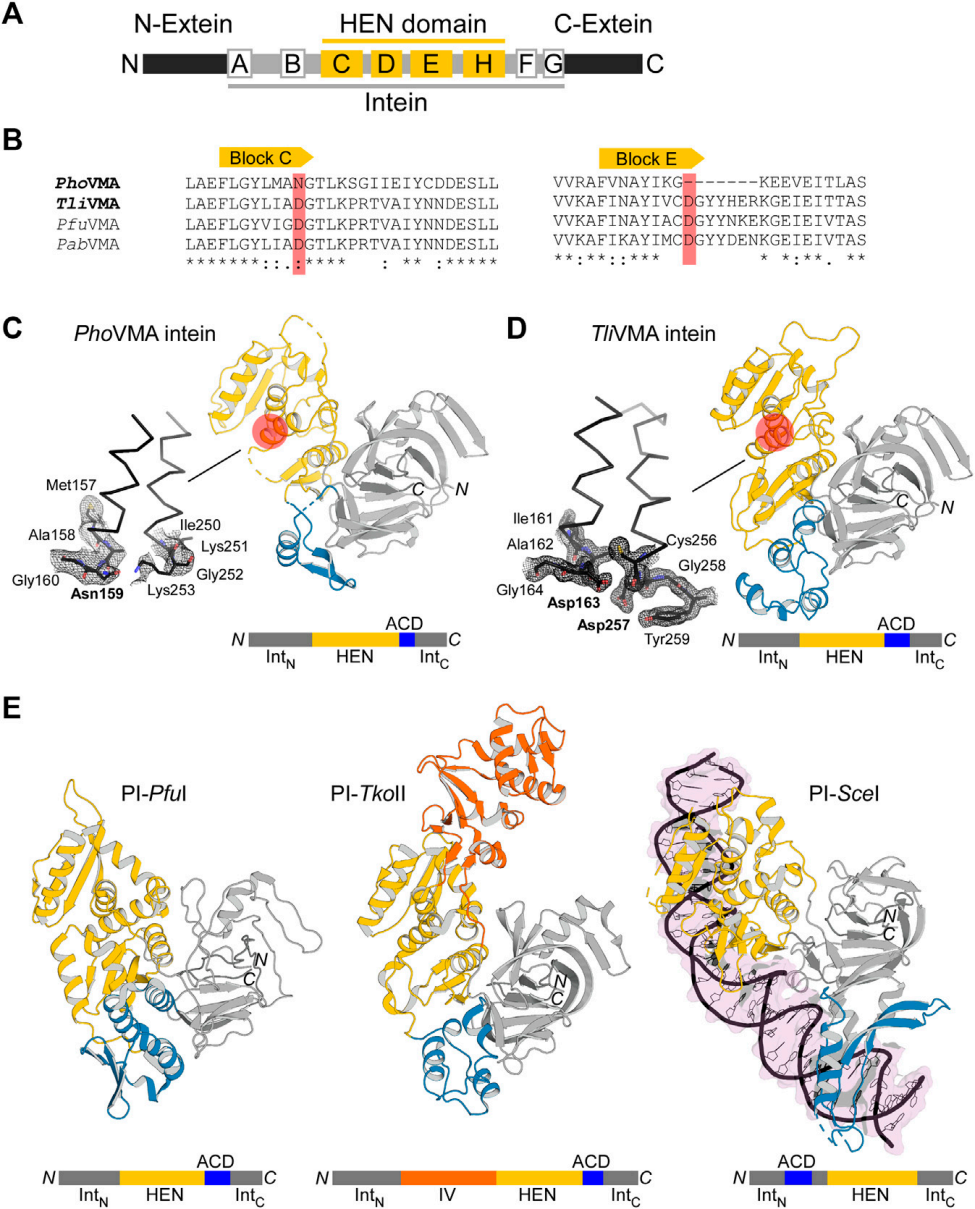
Structures of degenerated and active VMA inteins. **(A)** General domain organization and conservation in inteins. The HEN domain resides within the intein while the intein resides within a host protein (N- and C-exteins). Conserved sequence Blocks A–H are indicated. Host protein, black; intein, gray; HEN, yellow. **(B)** Sequence comparison around Blocks C and E corresponding to the active site-carrying LAGLIDADG helices of the HEN domains. Comparison of the intein orthologs of *Pyrococcus horikoshii* (*Pho*VMA), *Thermococcus litoralis* (*Tli*VMA), *Pyrococcus furiosus* (*Pfu*VMA), and *Pyrococcus abyssi* (*Pab*VMA). The position of the catalytic aspartates in Blocks C and E are highlighted in red. **(C)** Crystal structure of *Pho*VMA intein. **(D)** Crystal structure of *Tli*VMA intein. For **(C,D)**, the locations of the active sites are highlighted by red circles. The close-ups of the active sites are shown to the left with electron density maps at 1.0 σ contour level. **(E)** The previously reported three crystal structures of PI-*Pfu*I (PDB: 1dq3) (Ichiyanagi et al., 2000)(Ichiyanagi et al., 2000), PI-*Tko*II (PDB: 2cw7) (Matsumura et al., 2006), and PI-*Sce*I (PDB: 1lws) (Moure et al., 2002). In **(C–E)**, HINT, HEN, and ACD domains are colored in gray, yellow, and blue, respectively. PI-*Tko*II contains an additional domain IV indicated in orange. Int_N_ and Int_C_ indicate the N- and C-terminal parts of the HINT domain, which are separated by the HEN. The domain arrangement is schematically illustrated below each structure. *N* and *C* denote the termini.

To understand the molecular evolution of inteins, we are interested in elucidating three-dimensional structures of various inteins with a presumable HEN domain. The first intein was identified as an intervening sequence within the yeast vacuolar membrane ATPase (VMA), subunit A (Hirata et al., 1990). The majority of inteins among eukaryotes reside at the highly conserved insertion site within the Vacuolar ATPase (VMA-a insertion site) (Swithers et al., 2009). The extensively investigated VMA intein from *Saccharomyces cerevisiae* (*Sce*VMA) defines a proto-typical intein possessing homing endonuclease activity, also called PI-*Sce*I, as a rare cutting DNA endonuclease (Grindl et al., 1998). Whereas yeast inteins are inserted at the highly conserved insertion site (VMA-a site), archaeal inteins commonly target a region approximately 20-residue downstream of the VMA-a insertion site (VMA-b insertion site), located at the P-loop motif of ATPases (Swithers et al., 2009). The VMA intein from *P. horikoshii* (*Pho*VMA) consists of 376 amino acids, which is considerably smaller than canonical HEN-associated inteins, e.g., *Sce*VMA consisting of 454 residues but more similar to the size of the TFIIB intein from *Methanococcus jannaschii* (*Mja*TFIIB, 335 residues). The structure of the *Mja*TFIIB intein could previously not be determined together with the HEN domain by protein crystallography (Iwaï et al., 2017). Inteins share conserved amino acid sequence stretches designated as Blocks A-G (Pietrokovski, 1994; Perler et al., 1997) (Figure 2A). Blocks C, D, E, and H denote the HEN domain, out of which Blocks C and E represent the eponymous conserved LAGLIDADG helices bearing the acidic catalytic residues (Perler et al., 1997). The sequence alignment of the archaeal inteins also suggests that *Pho*VMA intein lacks homing endonuclease activity due to the absence of the active site residues in Blocks C and E (Figure 2B). We were successful in producing the *Pho*VMA intein and obtaining diffracting crystals. We solved the crystal structure of the *Pho*VMA intein at the 2.5 Å-resolution (Figure 2C; Supplementary Table S1). The crystal structure of *Pho*VMA intein revealed the typical HINT domain of thermophilic inteins, which contains a β-strand insertion and the HEN domain structure (Figure 2C) (Aranko et al., 2014). As expected from the primary structure, the *Pho*VMA intein lacks the presumed HEN active site residues in both usually conserved LAGLIDADG helices (Blocks C and E). It shows a partial truncation in Block E, hypothetically indicating progressive HEN degeneration (Figures 2A–C).

The length of inteins considerably varies from 123 to >1,000 residues due to various insertions such as HENs and sequence deletions (Green et al., 2018). Large intein sequences generally indicate the presence of an active or inactive nested HEN. Therefore, we were interested in elucidating the structures of other VMA inteins inserted at the same VMA-b site to reveal a possible structural basis directing inteins of diverse sizes to the same target insertion site within host genomes. Among VMA inteins inserted at the VMA-b insertion site, we could obtain crystals of the VMA intein from *Thermococcus litoralis* (*Tli*). The *Tli*VMA intein comprises 429 residues and is larger than the *Pho*VMA intein (376 residues) but similar to the size of PI-*Sce*I (454 residues). To prevent self-cleavages during protein production, we expressed both inteins in *E. coli* with alanine substitutions of the catalytic cysteines 1 (Cys1). We used the N-terminal small ubiquitin-like modifier (SUMO) fusion to facilitate protein purification of *Pho*VMA intein (Guerrero et al., 2015). However, *Tli*VMA intein required an N-terminal MBP fusion in addition to SUMO (H_6_-MBP-SUMO-*Tli*VMA intein) for successful soluble expression (Guerrero et al., 2015). Unlike *Pho*VMA intein with the presumably degenerated HEN domain, *Tli*VMA intein also required a high salt buffer composition, compensating for the lack of nucleic acids to maintain solubility after proteolytic removal of the fusion tag.

We solved the structure of *Tli*VMA intein at the 1.6 Å-resolution (Figure 2D, Supplementary Table S1). The crystal structures of the *Tli*VMA intein revealed a very similar overall structure as found in the *Pho*VMA intein, including the three-domain architecture known from the three other reported HEN-containing inteins (Figures 2C–E) (Duan et al., 1997; Ichiyanagi et al., 2000; Moure et al., 2002; Matsumura et al., 2006). The Hedgehog/Intein domains (HINT, gray) are composed of the N- and C-terminal fragments (Int_N_ and Int_C_) with the HEN domains (yellow) inserted into the common intein split-site located between the two pseudo-two-fold symmetrical units forming a horseshoe-like fold common to all HINT domains (Eryilmaz et al., 2014; Iwaï et al., 2017). The HINT domain of the *Tli*VMA intein also contains the β-strand extension found among thermophilic inteins (Figure 2C) (Aranko et al., 2014; Beyer et al., 2019; Hiltunen et al., 2021).

### The Differences Between *P. horikoshii VMA* and *T. litoralis VMA* Inteins

Not surprisingly, the HINT domains of *Pho*VMA and*Tli*VMA inteins show a virtually identical three-dimensional structure with a 86% sequence identity (Figure 2; Supplementary Table S4). The HINT domains connect to the first of the two LAGLIDADG helices of the HEN domains via unstructured loops of 28–32 residues, located distant from the DNA-binding interfaces. We have identified “accessory domains” (ACD, shown in blue) residing between the HEN domains and the C-terminal part of the HINT domains, where we observed the most notable differences. The striking contrast between both inteins is the divergence of their ACDs, showing the least structural homology between the two molecules (33% pairwise sequence identity, Figures 2C,D; Supplementary Figure S3). The 53-residue difference in the lengths between the *Pho*VMA and*Tli*VMA inteins can be mainly attributed to the difference in the ACDs. Even though ACDs at the intersections between HINT and HEN domain were identified in other reported HEN-associated inteins, their biological functions remain unclear (Figure 2E) (Duan et al., 1997; Ichiyanagi et al., 2000; Moure et al., 2002; Matsumura et al., 2006).

As for the nested HEN domains, the deletion in Block E in the *Pho*VMA intein (Figure 2B) causes a truncation of the second LAGLIDADG helix by one turn, thereby removing one of the catalytic aspartate residues (Figures 2B–D). Another obvious consequence of the degeneration in the *Pho*VMA intein appeared in the structure of the DNA-binding interfaces of the HEN mediated by two stretches of β-sheets, each originating from one copy of the two-fold pseudo symmetric LAGLIDADG elements (Supplementary Figures S1A,B) (Moure et al., 2002). The electrostatic surface potential of the HEN domains is very different between the two VMA inteins, which is in line with their binding to DNA fragments (see below).

Based on the three-dimensional structures, we deleted the HEN domain (residues 123-335 for *Pho*VMA intein and 123-388 for *Tli*VMA intein) and connected with an “NG” sequence linker, resulting in 165-residue *cis*-splicing *Pho*VMA^ΔHEN^ and *Tli*VMA^ΔHEN^ inteins. We modeled the structure of the two deletion variants with the RoseTTAFold software using the deep-learning algorithm (Supplementary Figures S1C,D) (Baek et al., 2021). Both structures appear identical with high confidence scores and an r.m.s.d. of 0.7 Å for 165 Cα atoms.

The *Pho*VMA^ΔHEN^ intein still retained the protein splicing activity, indicating that the HINT domain of *Pho*VMA intein is functionally independent of the nested HEN domain without having developed a mutualism (Supplementary Figure S1C) (Iwaï et al., 2017). However, the protein splicing activity of the *Tli*VMA^ΔHEN^ intein largely diminished, presumably because of the mutualism developed between the HINT and HEN domains (Supplementary Figure S1D) (Iwaï et al., 2017). Even though the two three-dimensional structures are predicted to be almost identical to the original HINT domain (Supplementary Figures S1C,D), the HEN domain of *Tli*VMA intein likely contributes to the protein splicing activity, as it has also been suggested for *Mvu*TFIIB intein (Iwaï et al., 2017).

### The HEN Domain of *Pho*VMA Intein Has Degenerated, and Its Activity Can Be Rescued

The primary structures and the three-dimensional crystal structures of the VMA inteins suggest that the HEN activity of *Pho*VMA intein has most likely degenerated during evolution and is inactive due to the lack of active site aspartate residues. However, *Tli*VMA intein probably carries a catalytically active HEN domain capable of binding to DNA and introducing DNA double-strand breaks (Figures 2A,B).

**FIGURE 3:**
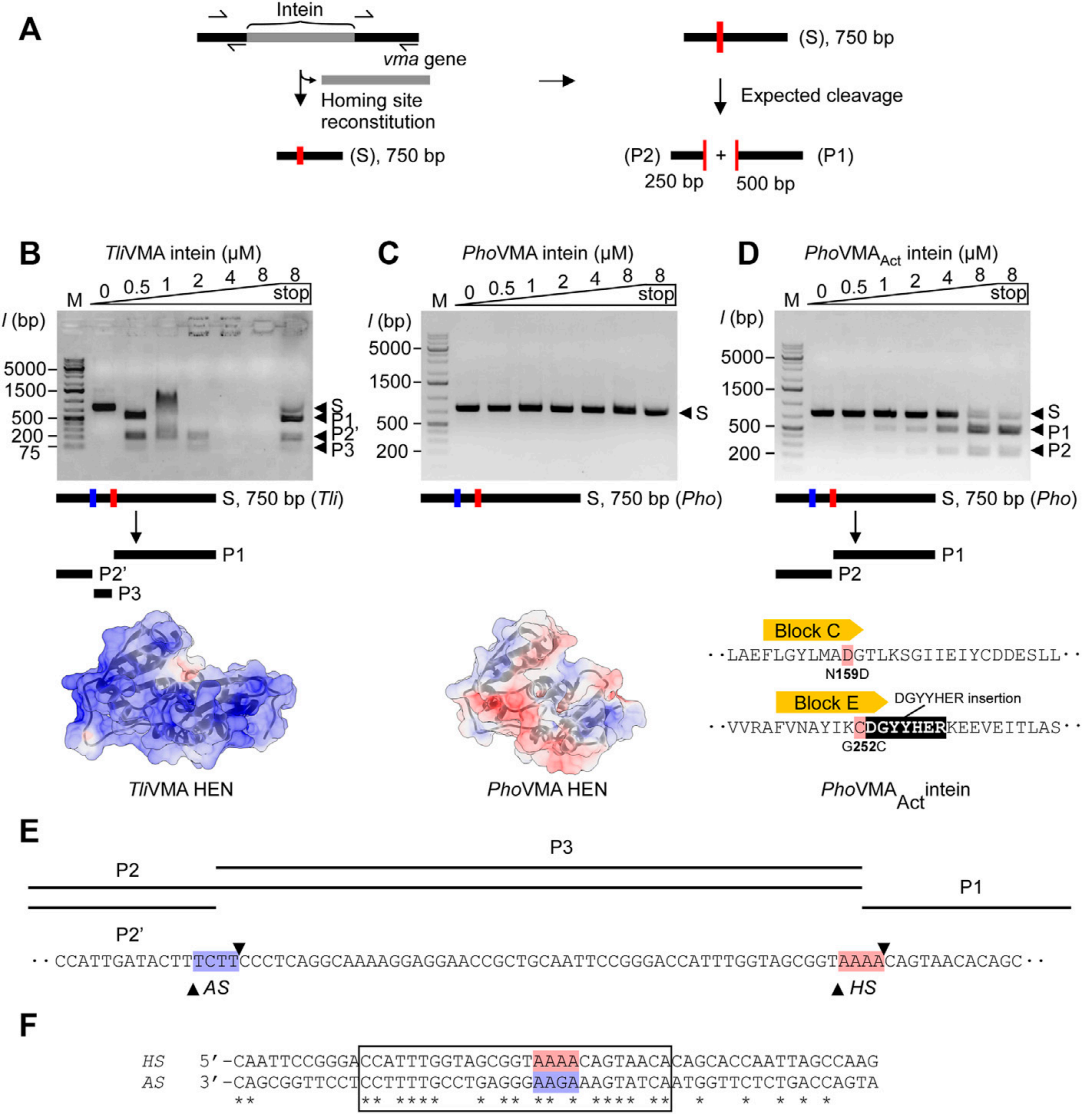
DNA-binding and cleavages of the theoretical homing sites by *Tli*VMA and *Pho*VMA intein variants. **(A)** PCR-based construction of linear DNA substrates from *Tli* and *Pho* genomic DNA and expected cleavage pattern. The homing site was reconstituted by deleting the intein coding sequence from the *vma* gene while adjusting the size to 750 bp asymmetrically harboring the homing sites to generate the expected 250- and 500-bp cleavage products. **(B)** DNA-binding and cleavage of the 750-bp *Tli* DNA substrate by incubation with increasing concentrations of *Tli*VMA intein at 80°C for 2 h. The electrostatic surface potential with an isoelectric point of 10.23 is shown below with a view of the DNA-binding interface. Positive, blue; neutral, white; negative, red. **(C)** Experiment as in **(B)** but using the 750-bp *Pho* DNA substrate and *Pho*VMA intein. The isoelectric point of the electrostatic surface potential is 5.45. The electrostatic surface potential model was generated using an alternative coordinate file without gaps in the HEN domain. **(D)** Activity test of the reactivated *Pho*VMA_Act_ intein. Experiment as in **(C)** but using *Pho*VMA_Act_ intein. The reconstitution of active site regions in the *Pho*VMA intein by grafting the sequence from Block C and E regions of the *Tli*VMA intein is illustrated below. In panels **(B–D)**, S, substrate; P1, 500-bp product, P2, 250-bp product, P2′, ∼200-bp product, P3, ∼50-bp product. M stands for the DNA ladder, “stop” indicates the addition of SDS-containing stop solution after incubation. **(E)** Distance of homing site (*HS*) and alternative site (*AS*) in the *T. litoralis* DNA substrate. Arrowheads indicate the positions where strand cleavage occurs. **(F)** The alignment of *Tli*VMA intein homing (*HS*) and alternative (*AS*, reversed) sites. A region of 27 bp with high sequence similarity between the *HS* and the reversed *AS* is indicated by a box. The central four base pairs where cleavage occurs are highlighted in red (*HS*) and blue (*AS*).

To experimentally validate these assumptions, we performed *in vitro* DNA-binding and cleavage studies. First, we generated DNA substrates containing the theoretical homing sites of the inteins, that is, the coding DNA sequence of the *Tli* and *Pho vma* genes without the intein coding region (Figure 3A). These reconstructed intein-less DNA fragments should resemble the allelic situation before invasion by the inteins (Figure 3A). We generated 750-bp linear double-strand DNA fragments asymmetrically harboring the reconstituted homing site by amplifying their respective sequences from the genomic DNA by PCR and tested the cleavage of the DNA fragments by the inteins. Indeed, we observed that *Tli*VMA intein cleaved its reconstituted homing site accompanied by a strong DNA binding affinity as indicated by an electrophoretic mobility shift (EMSA) of the substrate- and product-DNA fragments (Figure 3B). In contrast, *Pho*VMA intein was neither able to process its theoretical homing site, nor did it show any detectable DNA binding affinity (Figure 3C). Thus, the DNA substrates with the reconstituted homing sites validated our assumptions derived from the structures of *Tli* and *Pho* VMA inteins.

We attributed the catalytic inactivity of the HEN in *Pho*VMA intein to the loss of presumed active-site residues. The differences in the electrostatic surface potential distributions of the HEN domains between *Tli* and *Pho* VMA inteins further might support the weaker DNA-binding affinity of *Pho*VMA compared with *Tli*VMA intein (Figures 3B,C). Because the architecture of the HEN domain in *Pho*VMA intein was retained intact despite degenerative mutations and deletion, we wondered whether the nuclease activity of *Pho*VMA intein could be restored by protein engineering to reverse the evolutional process. To validate our hypothesis, we engineered the inactive *Pho*VMA intein by grafting the active sites in the LAGLIDADG helices from the sequences of the *Tli*VMA intein (Figures 2B, 3D). Indeed, the engineered *Pho*VMA intein (*Pho*VMA_Act_ intein) with the restored catalytic residues cleaved the DNA substrate containing the reconstituted homing site, albeit less efficiently not attaining the complete substrate digestion as observed with *Tli*VMA intein (*Tli*VMA intein, Figure 3B; *Pho*VMA_Act_ intein, Figure 3D).

### *Tli*VMA and *Pho*VMA_Act_ Inteins Differ in Homing Site Recognition

We designed and generated the DNA substrates for the DNA cleavage assay from *Tli* and *Pho* genomic DNA using PCR. Removing the intein coding sequences from the *vma* genes restored the theoretical homing site within a linear DNA of 750 bp containing the 250- and 500-bp fragments of the genomic sequences upstream and downstream of the reconstituted homing site, respectively (Figure 3A). The DNA cleavage at the homing site by the VMA inteins should produce 250- and 500-bp products. While the engineered *Pho*VMA_Act_ intein produced the expected two fragments (Figure 3D), *Tli*VMA intein exhibited an unexpected pattern of the products (Figure 3B). The disappearance of the DNA fragments at higher concentrations of *Tli*VMA intein without SDS-treated denaturation is presumably due to the strong affinity to the DNA molecule (“end-holding”). Interestingly, besides the expected 500-bp fragment, a product of ∼200 bp and a third one shorter than 75 bp appeared with *Tli*VMA intein. The analysis of the cleavage products by DNA sequencing revealed that *Pho*VMA_Act_ intein cleaved precisely at the expected homing site (Supplementary Figure S2A).

In contrast, *Tli*VMA intein cleaved at two different sites. One site was indeed at the theoretical homing site (*HS*) with the central four base pairs of the sequence 5′-AAAA-3′, while the other alternative site (*AS*) is located 52 bp upstream of the reconstituted homing site and contains the central sequence 5′-TCTT-3′ (Supplementary Figure S2B). We assume that recognition and cleavage of the *AS* by *Tli*VMA intein occur on the opposite strand of the *HS*. The sequence on the opposite strand corresponds to the sequence of 5′-AAGA-3′, reminiscent of the reconstituted homing site of *Pho*VMA_Act_ intein (Figures 3E,F; Supplementary Figure S2C) and bearing a single substitution to the central four base pairs of the homing site of *Tli*VMA intein. Indeed, the alignment of the DNA sequence against the reverse strand of the alternative site revealed a striking 63% identity encompassing a 30 bp region surrounding the two cleavage sites (Figure 3F). Overall, the DNA substrates reconstituted from *Tli* and *Pho* genomic DNA have sufficient similarity to assume that both contain the *AS* next to the *HS* (Supplementary Figure S2C). However, *Pho*VMA_Act_ intein could exclusively process the homing site (*HS*), leaving the *AS* unaffected (Figure 3D). We could conclude that the activated *Pho*VMA_Act_ intein is more specific toward recognizing the reconstituted homing site (*HS*) despite its lower affinity.

### The *Tli*VMA Intein Accessory Domain Lowers DNA Cleavage Specificity

The lengthy DNA sequences recognized by homing endonucleases (HENs) attracted protein engineering of HENs for genomic application because the high specificity of HENs could facilitate various *in vitro* and *in vivo* applications (Stoddard, 2011). However, the number of HENs that recognize different DNA sequences which could be used for broad applications is small. Although dozens of intein structures have been deposited to the protein data bank (PDB), only three of those contain a nested HEN domain. Moreover, exclusively the intein structure of PI-*Sce*I from *Saccharomyces cerevisiae* was elucidated as the DNA/intein complex (Duan et al., 1997; Moure et al., 2002). The limited structural information of HEN-associated inteins hinders our understanding of inteins as site-specific DNA endonucleases, impeding further development of HEN-associated inteins by protein engineering as genetic engineering tools. Other reported HEN-associated intein structures are archaeal inteins from *Thermococcus kodakaraensis* (PI-*Tko*II) (Matsumura et al., 2006) and *Pyrococcus furiosus* (PI-*Pfu*I) (Ichiyanagi et al., 2000). Just like the VMA inteins from *T. litoralis* and *P. horikoshii*, these inteins have an accessory domain (ACD) in addition to HINT and HEN domains (Figure 2D). Furthermore, in the case of PI-*Tko*II, an additional domain, termed domain IV, was reported (Matsumura et al., 2006).

It is believed that ACDs in HEN-associated inteins might generally contribute to interactions with DNA. For PI-*Sce*I, where the ACD is referred to as DNA recognition region (DRR), this role has been demonstrated, although the location of the ACD in PI-*Sce*I is different from other reported HEN-associated intein structures (Figures 2C–E; Supplementary Figure S3). The ACD (DDR) can be seen as an insertion into the HINT domain rather than a connection of HEN and HINT domains (Moure et al., 2002) (Figure 2E; Supplementary Figure S3). However, HENs also exist free-standing without being embedded in inteins or introns. They are known to be among the most sequence-specific endonucleases due to their relatively long sequence recognition motif (Chevalier and Stoddard, 2001). Some of such HENs do not contain ACDs. Thus, it remains elusive why some HEN-associate inteins require ACDs and cannot define sufficient DNA sequence specificity with their intrinsic DNA recognition capability.

**FIGURE 4:**
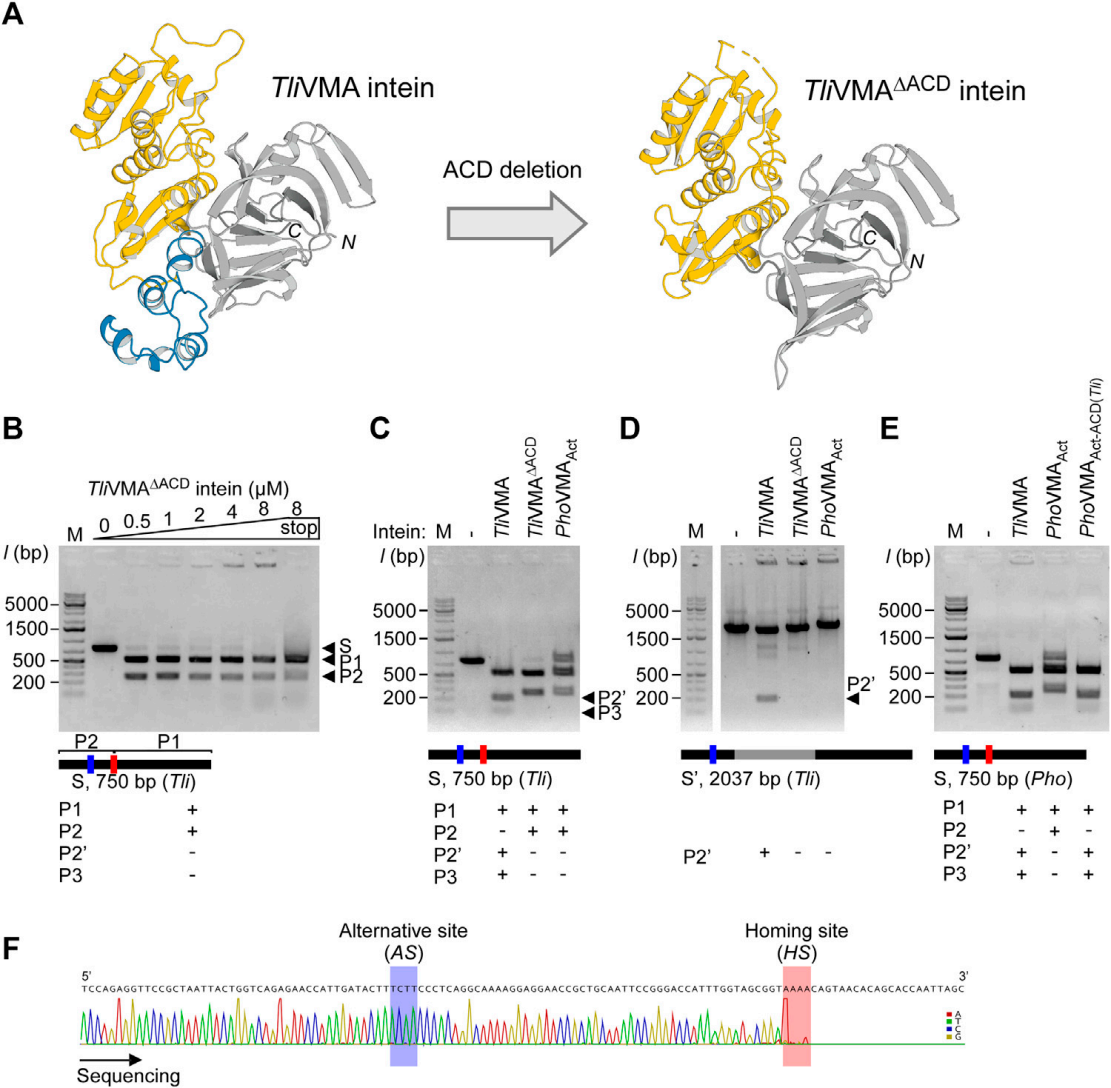
Deletion and grafting of the ACD in the *Tli*VMA intein. **(A)** The crystal structures of *Tli*VMA intein (left) and *Tli*VMA^ΔACD^ intein (right) without ACD, connecting the HEN domain and the C-terminal part of the HINT domain. HEN, ACD, and HINT are colored in yellow, blue, and gray, respectively. **(B)** DNA-binding and cleavages of the reconstituted 750-bp DNA fragment with the homing site from *Thermococcus litoralis* genome (*Tli*) by *Tli*VMA^ΔACD^ intein. The cleavages were analyzed on agarose gels after the incubation with increasing concentrations at 80°C for 2 h. **(C)** Comparison of DNA cleavages of the reconstituted 750-bp *Tli* DNA fragment by *Tli*VMA, *Tli*VMA^ΔACD^, and *Pho*VMA_Act_ inteins. **(D)** Comparison of the DNA cleavages of the 2037 bp DNA fragment, including the *Tli*VMA intein coding sequence at the homing site by the three inteins as in C. This fragment lacked the reconstituted homing site (*HS*) due to the *Tli*VMA intein coding sequence, hence only possessed the alternative site (*AS*). **(E)** DNA cleavages of the 750-bp *Pho* DNA fragment by the reactivated *Pho*VMA_Act_ intein with the ACD from *Tli*VMA intein (*Pho*VMA_Act-ACD(*Tli*)_) and the comparison with *Tli*VMA and *Pho*VMA_Act_ inteins. In **(B–E)**, M stands for the DNA ladder, “stop” indicates the addition of SDS-containing stop solution after incubation. The migration height for the 750-bp substrate (S) and the 500 bp (P1) and 250 bp (P2) products are indicated in **(B)**, P2’ (200 bp), and P3 (50 bp) are shown in **(C,D)**. **(F)** The Sanger sequencing chromatogram of the 250-bp cleavage product generated by the *Tli*VMA^ΔACD^ intein lacking the ACD.

To investigate the structural and functional roles of ACDs in HEN-associated inteins, we decided to delete the ACD region from the *Tli*VMA intein based on the three-dimensional structure. We could also validate our deletion design by determining the crystal structure of the deletion variant, termed *Tli*VMA^ΔACD^ intein (Figure 4A; Supplementary Table S1). The crystal structure of *Tli*VMA^ΔACD^ intein confirmed that the deletion of the ACD did not influence the HINT and HEN domain folds, nor their relative orientation toward each other (Figure 4A). In the DNA cleavage and binding assays, *Tli*VMA^ΔACD^ intein similarly cleaved the same substrate as the wild-type *Tli*VMA intein did, albeit with reduced DNA-binding (Figures 3B, 4B). To our surprise, the deletion of the ACD from the *Tli*VMA intein changed the cleavage profile. The substrate cleavage profile by *Tli*VMA^ΔACD^ intein resembled that of the *Pho*VMA_Act_ intein, producing two main products as opposed to three products generated by *Tli*VMA intein (Figure 4C). The DNA sequencing chromatogram of the smaller cleavage product generated by *Tli*VMA^ΔACD^ supported that *Tli*VMA^ΔACD^ intein did not cleave the alternative site as observed for the wild-type *Tli*VMA intein, similar to the digestion pattern of the *Pho*VMA_Act_ intein (Figure 4F). Moreover, we found that *Tli*VMA intein cleaved the reconstituted DNA substrate from the *Pyrococcus horikoshii* (*Pho*) genome at the alternative cleavage site (Figure 4E).

Similarly, *Pho*VMA_Act_ intein was able to digest the homing site within the *Tli* genome, albeit less efficiently as its cognate homing site (Figure 4C; Supplementary Figure S4). This cross-activity between *Tli*VMA and *Pho*VMA_Act_ inteins is presumably due to the close homology between the two substrate sequences created from *Pho* and *Tli* genome (Supplementary Figures S2C, S6A). The deletion of ACDs suggests that ACDs could play critical roles in increasing the cleavage specificity of HEN-associated inteins as well as making them more promiscuous by adding the capability to recognize an alternative cleavage site.

Next, we were interested in how the ACD in *Tli*VMA intein influences the DNA recognition specificity. We speculated two possible scenarios: direct recognition of the alternative site sequence mediated by the ACD or indirect recognition via a cooperative binding effect. The binding of the intein to the homing site could guide the recognition of the alternative site separated by only 52 bp from the homing site by cooperative domain interaction with a second intein molecule involving the ACD. The DNA substrate containing only the alternative cleavage (*AS*) site (intein coding sequence remained inserted into the homing site) indicated that only *Tli*VMA intein bearing the ACD was capable of digesting the DNA substrate, whereas *Tli*VMA^ΔACD^ intein was not (Figure 4D). These results revealed that the *Tli*VMA intein cleaved the alternative site (*AS*) independent of the homing site (*HS*) but depended on the presence of the ACD domain. We also performed DNA-binding tests using the isolated ACD domain of the *Tli*VMA intein and revealed that the ACD seemingly does not contribute to the overall DNA affinity (Supplementary Figure S5).

To further validate the role of the ACD in *Tli*VMA intein as a modulator of the DNA recognition responsible for the alternative site, we tested whether grafting of the ACD in *Pho*VMA_Act_ intein from *Tli*VMA intein would confer alternative site recognition. We thus created *Pho*VMA_Act-ACD(*Tli*)_ intein having the grafted ACD from *Tli*VMA intein (ACD (*Tli*)). Indeed, *Pho*VMA_Act-ACD(*Tli*)_ could process both homing and alternative sites of the DNA substrate generated from the *Pho* genome, reminiscent of the cleavage profile produced by the *Tli*VMA intein (Figure 4E). Furthermore, the swapping of the ACD rendered *Pho*VMA_Act-ACD(*Tli*)_ intein more efficient in processing the DNA substrate without altering the apparent overall DNA affinity (Figure 3D; Supplementary Figure S6B). The weaker activity of *Pho*VMA_Act-ACD(*Tli*)_ intein also allowed resolving a preferentiality of the homing site over the alternative site as the latter required a higher enzyme concentration (Supplementary Figure S6B).

**FIGURE 5:**
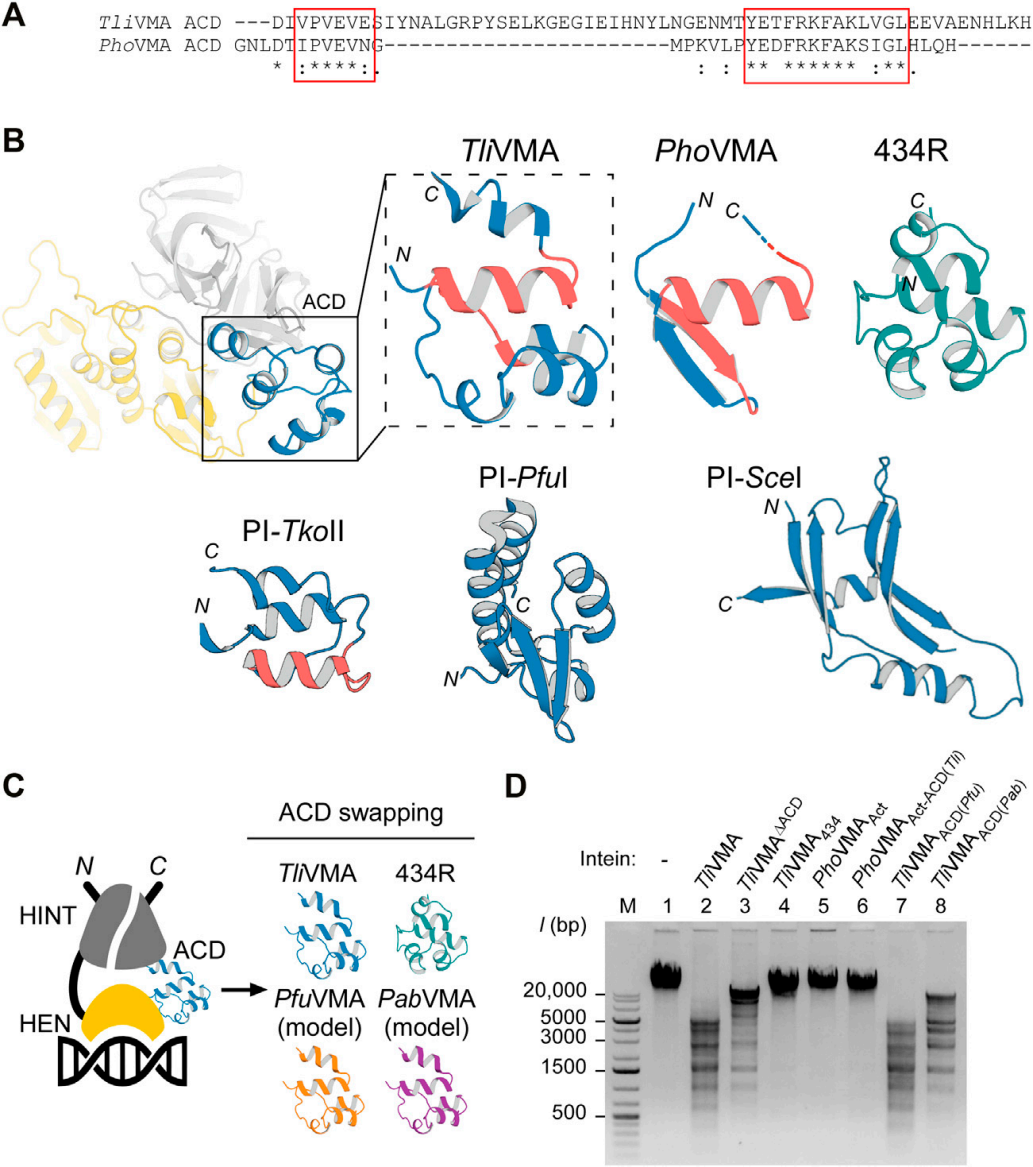
Effect of swapping ACDs in the VMA inteins on DNA recognition. **(A)** Primary structure comparison of ACDs in *Tli* and *Pho* VMA inteins. Regions with high similarity are highlighted with red rectangles. **(B)** The ACD structures of *Tli* and *Pho* VMA inteins and a comparison with the bacteriophage 434 repressor (434R, PDB: 2or1) (Aggarwal et al., 1988) and the ACDs of PI-*Tko*II (PDB: 2cw7), PI-*Pfu*I (PDB: 1dq3), and PI-*Sce*I (PDB: 1lws). The regions highlighted with red rectangles in **(B)** are colored in red. **(C)** Schematic illustration of the swapping experiments using different ACDs. The ACD in the *Tli*VMA intein was replaced with the respective ACDs from the VMA intein of *P. furiosus* (*Pfu*), *P. abyssi* (*Pab*), or the 434-bacteriophage repressor domain. **(D)** DNA cleavages of λ-phage DNA using *Tli* and *Pho* VMA intein variants carrying different ACDs. Cleavage of λ-phage DNA was tested by overnight incubation with the indicated intein variants at 80°C. Agarose gel analysis of the digestion reactions. Lane 1, λ-phage DNA (48k bp) without intein; lane 2, the wild-type *Tli*VMA intein; lane 3, *Tli*VMA intein with deletion of ACD (*Tli*VMA^ΔACD^); lane 4, *Tli*VMA intein with phage 434 repressor as ACD (*Tli*VMA_434_); lane 5, reactivated *Pho*VMA_Act_ intein; lane 6, the reactivated *Pho*VMA intein with ACD from *Tli*VMA intein (*Pho*VMA_Act-ACD(*Tli*)_); lane 7, *Tli*VMA with ACD from *Pfu*VMA (*Tli*VMA_ACD(*Pfu*)_); lane 8, *Tli*VMA intein with ACD from *Pab*VMA (*Tli*VMA_ACD(*Pab*)_). M stands for the DNA size ladder.

The crystal structures of *Tli* and *Pho*VMA inteins, inserted at the same VMA-b insertion site of their host proteins, revealed a notable structural difference in their ACDs, largely deviating from each other (Figures 2C, 5A,B). The structural difference prompted us to investigate the functional role of ACDs. Our results demonstrated that the ACD in *Tli*VMA intein induced a second cleavage site in addition to the theoretical homing site (Figure 4C). Interestingly, engineering the reactivated *Pho*VMA intein by grafting the ACD from *Tli*VMA intein triggered cleavage at the alternative site (*AS*) adjacent to the homing site (*HS*), suggesting that the ACD is responsible for the cleavage at the *AS* (Figure 4E). The ACD of *Tli*VMA intein strongly resembles the helix-turn-helix motif common for many DNA binding proteins, such as transcriptional regulator proteins (Figures 5A,B; Supplementary Table S5) (Anderson et al., 1981; Brennan and Matthews, 1989). The homology to DNA binding proteins suggests that ACDs mediate contacts with the DNA substrate.

Next, we wanted to test whether *Tli*VMA intein is a promiscuous endonuclease and could cut unrelated substrates. We, therefore, tested digestion of λ-phage DNA by incubating overnight with *Tli*VMA intein or *Tli*VMA^ΔACD^ intein lacking the ACD (Figures 5C,D). To our surprise, we identified multiple cleavages in line with our observations using the model DNA substrates generated from *T. litoralis* genomic DNA. Furthermore, similar to our model DNA substrate, deletion of the ACD in *Tli*VMA intein indeed reduced cleavage of λ-phage DNA, supporting our hypothesis that the ACD renders the intein endonuclease more promiscuous. In contrast, the activated *Pho*VMA_Act_ intein with the endogenous ACD and the activated *Pho*VMA_Act-ACD(*Tli*)_ with the grafted ACD from *Tli*VMA intein (ACD (*Tli*)) did not produce any detectable λ-phage DNA cleavage, presumably due to the much lower affinity to the DNA substrate (Figures 3D, 4E, 5D).

We wondered how ACDs from other homologous inteins and an unrelated DNA-binding domain of the bacteriophage 434 repressor (434R) would affect cleavages of λ-phage DNA by *Tli*VMA intein (Aggarwal et al., 1988). We engineered *Tli*VMA intein by ACD-swapping the 434R domain and found that the engineered *Tli*VMA intein decreased λ-phage DNA processing. However, we could still detect some extent of cleavages (Figure 5D). Replacing the ACD in*Tli*VMA intein with an ACD from the more related inteins like VMA inteins from *P. furiosus* (*Pfu*VMA) and *P. abyssi* (*Pab*VMA) had a milder effect on the cleavage of λ-phage DNA (Figure 5D). Whereas the *Tli*VMA intein variant carrying the ACD from *Pfu*VMA intein (*Tli*VMA_ACD(*Pfu*)_) produced a restriction pattern very similar to the wild-type *Tli*VMA intein, the variant with the ACD from *Pab* (*Tli*VMA_ACD(*Pab*)_) exhibited a less similar pattern (Figure 5D). The difference in the digestion profiles might arise from the fact that the ACD from *Pfu*VMA intein has eight mutations, while the ACD from *Pab*VMA intein contains 12-residue changes relative to the 55-residue region of the ACD in the *Tli*VMA intein. Interestingly, replacing the ACD in the *Tli*VMA intein with an unrelated DNA binding domain of phage 434R nearly abolished the cleavage of λ-phage DNA by the *Tli*VMA intein (*Tli*VMA_434_), indicating that grafting of 434R might disrupt the functional structure completely or create steric hindrances due to the poor protein engineering.

## Discussion

Homing endonucleases as rare cutting DNA endonucleases have sparked great interest in gene targeting and genome engineering (Stoddard, 2011). Currently, four classes of targetable DNA cleavage enzymes exist: zinc-finger nucleases (ZFNs), transcription activator-like effector nucleases (TALENs), CRISPR/Cas RNA-guided nucleases (RGNs), and LAGLIDADG homing endonucleases (LHEs), the latter also termed “Meganucleases.” These enzymes can assist in targeted gene modification (Carroll, 2014). Engineering of rare cutting DNA endonucleases with novel desired recognition sites could open a myriad of *in vitro* and *in vivo* applications targeting specific DNA sequences. Whereas the modular architectures of TALENs and ZFNs facilitate their protein engineering attempts to recognize novel sequences (Maeder et al., 2008; Cermak et al., 2011), LAGLIDADG-type homing endonucleases (LHEs) have been the most difficult enzymes to engineer for altered DNA recognition (Taylor et al., 2012).

In this study, we determined the crystal structures of two archaeal inteins inserted at the same VMA-b site, revealing their molecular architecture consisting of HINT, HEN, and ACD. We found that the three-dimensional structures of ACDs were highly diverse among the five solved three-dimensional structures of inteins with nested HEN domains. Moreover, two ACDs from *Tli*VMA intein and PI-*Tko*II resemble typical DNA-binding proteins containing the helix-turn-helix motif (Figures 2D,E). The modular structures of the HEN-containing inteins motivated us to engineer the nested HEN-associated inteins with altered DNA specificities for cleaving novel target sites by engineering the ACDs. We originally assumed that the presence of the ACD provided a higher specificity by additional DNA binding mediated by the ACD.

Contrary to our expectation, the deletion of the ACD from *Tli*VMA intein and grafting of the ACD from *Tli*VMA intein to *Pho*VMA intein indicated that the ACD enables recognizing an additional cleavage site (*AS*), thereby rendering the homing endonuclease domain more promiscuous (Figures 4C,E). However, grafting of ACDs from other archaeal VMA inteins and an unrelated phage DNA binding domain resulted in different digestion profiles of λ-phage DNA. Protein engineering of ACDs suggests the potential of HEN-associated inteins as a scaffold for creating novel meganucleases capable of recognizing novel target sites. Further detailed characterization of DNA recognition mechanisms by HEN-associated inteins could open the possibility to develop novel reagents with modulated DNA recognition specificities (Pâques and Duchateau, 2007; Carroll, 2014).

**FIGURE 6:**
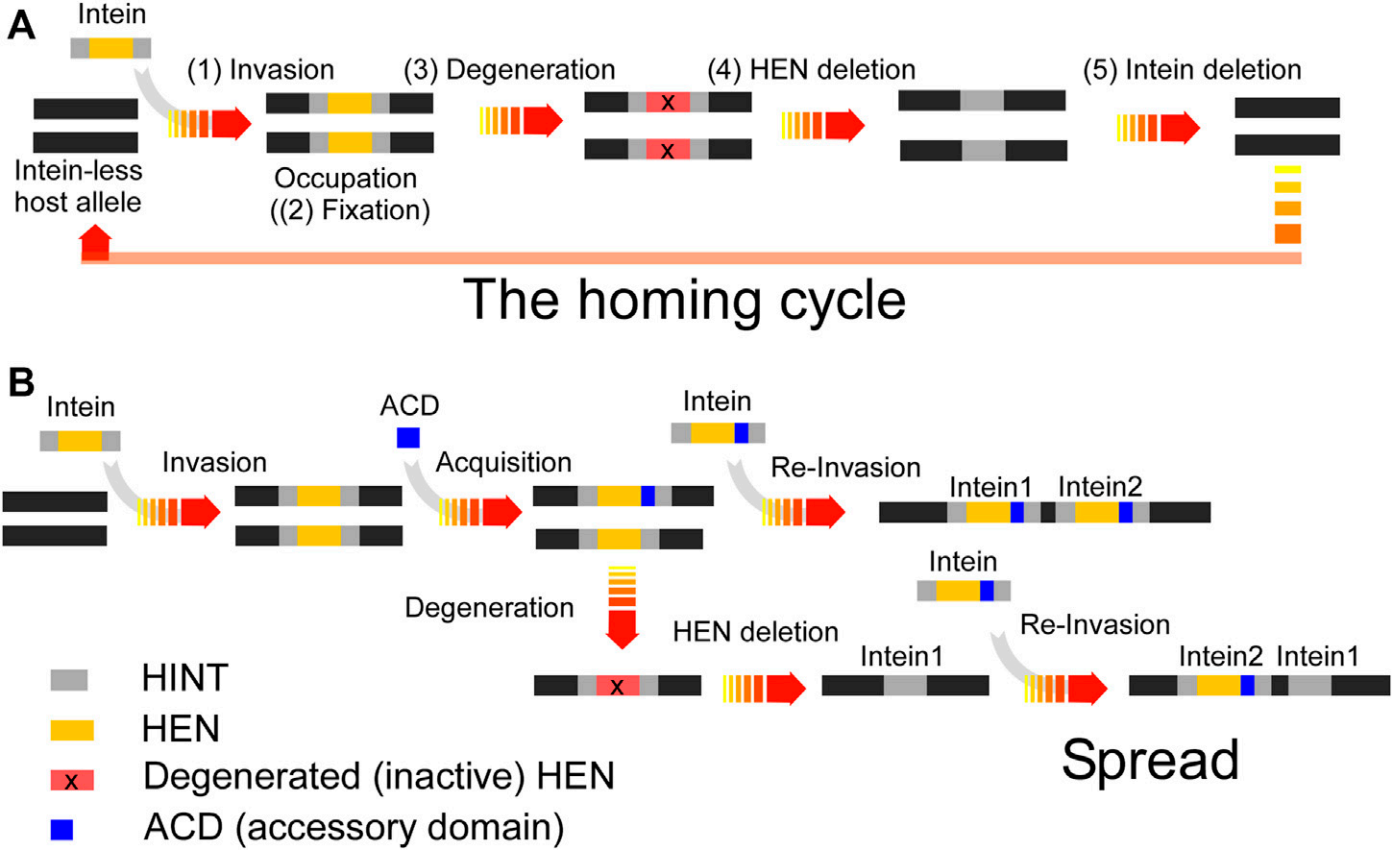
**(A)** The intein homing cycle model. The intein homing cycle model starts with the (1) Invasion of a HEN-containing intein via horizontal gene transfer followed by (2) Fixation into vacant alleles within the population. Depletion of HEN homing sites causes (3) Degeneration of the HEN due to accumulation of mutations tolerated by the lack of selection. Degenerated HENs are prone to (4) Deletion, rendering the intein incapable of competing with intein-free alleles, which might cause (5) Intein-loss upon interbreeding with strains providing an intein-free allele. **(B)** Intein spread model. Inteins might obtain an ACD modulating the HEN specificity, e.g., via changes in the ACD which can lower the HEN specificity to find novel insertion sites. Thus, the acquisition of a diverse ACD provides a spreading mechanism to prevent degeneration and extinction.

Inteins do not impact the host protein function because protein-splicing produces intact functional host proteins by self-excision of the inteins. Inteins, therefore, are found inserted into essential enzymes such as Vacuolar-type ATPase to ensure their selection. Abrogated inteins that accumulated mutations could result in inactive host proteins detrimental to the host organism. Therefore, protein splicing is required for the integrity of host proteins and establishes the selection. The homing endonuclease activity of inteins, however, is only required for invasion. Once the intein element occupies all target sites and is fixed in the population, the homing endonuclease activity degenerates and eventually becomes extinct, establishing the homing cycle (Figure 6A) (Burt and Koufopanou, 2004). In some inteins, HENs have developed a mutualism with HINT by making HINT dependent on the presence of the HEN scaffold for protein splicing (Iwaï et al., 2017). However, the mutualism between HINT and HEN could only slow down the eventual loss of HENs.

Our studies on ACDs in archaeal VMA inteins suggest that ACDs play an essential role in directing inteins to new alternative homing sites by acquiring diverse ACDs, presumably to avoid the extinction of HEN and HEN-associated inteins (Figure 6B). We hypothesize that HEN-associated inteins obtain an ACD from other genes such as transcription factors containing DNA-binding domains by yet unknown mechanisms to avoid the fixation (Figure 6A). The observed diversity in the structures of ACDs implies the divergent evolution and might support our hypothesis. Moreover, in nature, many genes host multiple inteins. For example, DNA polymerase from *Thermococcus kodakaraensis* hosts the two inteins PI-*Tko*I and PI-*Tko*II, separated by 85 amino acid residues in the host protein (Ichiyanagi et al., 2000). Cell division control protein 21 (CDC-21) in *Pyrococcus abyssi* also contains two mini-inteins separated by 48 amino-acid residues (Beyer et al., 2019). Thus, the prevalence of genes harboring multiple inteins in nature could support our hypothesis that inteins exploit ACDs for expanding the homing site to spread. However, there might still be other unknown advantages of having alternative cleavage sites by HEN-associated inteins (Figure 6B).

The structural basis of DNA recognition by HEN-associated inteins still awaits experimental elucidation of the high-resolution structure of DNA/inteins complexes. Such structural information of various HEN-associated inteins could shed light on the evolutionary histories of individual inteins and open a new avenue to develop a novel genetic engineering tool, which is smaller than RNA-guided nucleases for biotechnological applications.

## Materials and Methods

### Molecular Cloning, Protein Production, and Purification

All plasmids, oligonucleotides, and synthetic DNA substrate molecules used in this study are described in Supplementary Table S2. All recombinant proteins were produced in the *E. coli* strain T7 Express (New England Biolabs, USA). Expression details are given in Supplementary Table S3. All inteins carry a substitution of the catalytic cysteine 1 to alanine (C1A) to enable purification as fusion proteins except for those used in protein splicing tests. Residue numbering starts with 1 for this catalytic intein amino acid position and proceeds toward the C-terminus. Intein preceding residues are given negative indices.

Expression cultures were harvested by centrifugation at 4,700*g* for 10 min, 4°C. Pelleted cells from 1 or 2 L cultures were lysed in buffer A (50 mM sodium phosphate, pH 8.0, 300 mM NaCl) using continuous passaging through an EmulsiFlex-C3 homogenizer (Avestin, Canada) at 15,000 psi, 4°C for 10 min. Lysates were cleared by centrifugation at 38,000*g* for 60 min, 4°C. Proteins were purified in two steps using 5 ml HisTrap HP columns (GE Healthcare Life Sciences, USA) as previously described, including the removal of the hexahistidine tag and MBP and SUMO fusion domains (Guerrero et al., 2015).

After proteolytic removal of the fusion domains using Ubiquitin-like-specific protease 1 (UlpI), thermostable *Pho*VMA_Act_ intein (pHBRSF067) and *Tli*VMA^ΔACD^ intein (pHBRSF075) were heat fractionated at 80°C for 20 min before application to the Ni^2+^-NTA HisTrap column. After purification, all proteins were dialyzed overnight at 8°C against the following buffers: *Tli*VMA intein (pHBRSF063) was first dialyzed against 10 mM Tris-HCl pH 8.0, resulting in precipitation of the target protein. Precipitated protein was resolubilized by the addition of 500 mM KCl followed by dialysis against 10 mM Tris-HCl pH 8.0, 400 mM KCl. *Tli*VMA^ΔACD^ intein was treated like *Tli*VMA intein, but 800 mM KCl was used for resolubilization, and the last step of dialysis was omitted. *Pho*VMA intein (pCARSF54) was dialyzed against deionized water. *Pho*VMA_Act_ and *Pho*VMA_Act-ACD(*Tli*)_ intein (pHBRSF079) were dialyzed against 10 mM Tris-HCl pH 8.0, 300 mM KCl. The isolated ACD of *Tli* (pHBRSF082) was dialyzed against 20 mM Tris-HCl pH 8.0, 300 mM KCl. The *Tli*VMA intein variants containing exchanged ACDs (pHBRSF083, *Pfu*VMA ACD; pHBRSF084, *Pab*VMA ACD; pHBRSF161, 434-repressor domain) were purified like the *Tli*VMA intein (pHBRSF063). After proteolytic removal of the H_6_-MBP-SUMO purification tag, sample and purification buffers were supplemented with 350 and 200 mM KCl, respectively. After dialysis against salt-free buffers, the three proteins were resolubilized by addition of 475, 600, and 700 mM KCl, respectively.

Proteins were subsequently concentrated using Macrosep^®^ Advance Centrifugal Devices 10K MWCO (PALL). For enzymatic assays, proteins were diluted to 50 µM with 50 mM Tris-HCl pH 8.0, 300 mM KCl, 10% (v/v) glycerol, 1 mM dithiothreitol (DTT) and stored at −80°C for further use.

### Crystallization, Data Collection, and Structure Solution

All diffracting crystals were obtained at room temperature using the sitting drop vapour diffusion method by mixing 100 nL concentrated *Pho*VMA, *Tli*VMA, and *Tli*VMA^ΔACD^ inteins with 100 nL of the following mother liquors: *Pho*VMA, (100 mM HEPES pH 8.0, 5 mM cadmium chloride, 5 mM magnesium chloride, 5 mM nickel (II) chloride, 10% (w/v) polyethylene glycol (PEG) 3,350); *Tli*VMA, (100 mM magnesium formate, 15% (w/v) PEG 3350); *Tli*VMA^ΔACD^, (100 mM HEPES pH 7.5, 70% (v/v) 2-methyl-2,4-pentanediol (MPD)). Data were collected at beamline i04 (*Pho*VMA and *Tli*VMA^ΔACD^ inteins) at Diamond Light Source (Didcot, United Kingdom) and beamline ID30A-1 (MASSIF-1, *Tli*VMA intein) at ESRF (Grenoble, France) (Bowler et al., 2015). Data were processed using XDS (Kabsch, 2010). Structures were solved by molecular replacement (MR) starting from a *Pho*VMA intein search model generated using SWISS-MODEL (Arnold et al., 2006) with the intein homing endonuclease II of *Thermococcus kodakarensis* DNA polymerase (PDB: 2cw7) as a template. Initial models were obtained using the MR pipeline of Auto-Rickshaw (Panjikar et al., 2005) and ARP/wARP (Langer et al., 2008). Model-building and refinement were performed with COOT (Emsley et al., 2010) and PHENIX (Adams et al., 2002). The obtained structures were validated with Molprobity (Chen et al., 2010). Please refer to the Supporting Materials and Methods for a detailed description. Figures presenting three-dimensional coordinates were generated using PyMOL (The PyMOL Molecular Graphics System, Version 2.2.0, Schrödinger, LLC.). Electrostatic surface distributions were calculated and visualized using UCSF Chimera 1.13.1 (Pettersen et al., 2004) with ABS 1.3 (Jurrus et al., 2018) after model preparation with PDB2PQR 2.2.1 (Dolinsky et al., 2004) using a PARSE force field. Sequence alignments were performed with Clustal Omega 1.2.4. (Sievers et al., 2011).

### DNA-Cleavage Analysis by the Inteins

If not indicated otherwise, electrophoretic mobility shift and enzymatic cleavage assays were performed in a total volume of 10 µL in 10 mM Tris-HCl pH 8.0, 100 mM KCl, 10 mM MgCl_2_, 1 mM DTT using 0.05 µM (750 bp), or 0.01 µM (2037 bp) linear dsDNA substrates containing the desired intein homing site (Supplementary Table S2). Typically, reactions contained 0.5 µM of *Tli*VMA- or 8 µM of *Pho*VMA-derived inteins and were incubated at 80°C for 2 h. Where indicated, reactions were stopped by the addition of 1 µL endonuclease stop solution (5% (w/v) SDS, 250 mM EDTA, 100 mM Tris-HCl pH 7.5). Mobility shifts and cleavage products were visualized on 1.2% agarose gels.

For the determination of HEN cleavage sites, 1.5 µg of substrate DNA were digested overnight using the respective endonuclease with the above-described buffers, temperature, and concentrations. For the *Tli*VMA intein, a stop solution was used to dissociate the HEN from the restriction products. Products were gel-purified and sequenced via Eurofins Genomics GmbH using the exterior oligonucleotides as used for the generation of the DNA substrates (Supplementary Table S2). For the digestion of λ-phage DNA, 1 µg substrate was incubated overnight with the indicated intein variants as described above.

### *In Vivo* Protein *Cis*-Splicing Assays

Protein *cis*-splicing of intein variants was tested by expressing the indicated intein variants flanked by two B1 domains of the IgG-binding protein G in 5 ml cultures of *E. coli* strain T7 Express (New England Biolabs, USA) and purified using immobilized metal affinity chromatography as described elsewhere (Beyer et al., 2020). The used plasmids are listed in Supplementary Table S2. The experiments were performed at 30–37°C and the expression period lasted 3–4 h. Protein splicing was analyzed by SDS-PAGE using 16.5% gels and Coomassie Blue staining.

## Data Availability

The datasets presented in this study can be found in online repositories. The names of the repository/repositories and accession number(s) can be found in the article/Supplementary Material. Atomic coordinates and structure factors for the reported crystal structures have been deposited with the Protein Data bank under accession numbers 7QSS, 7QST, and 7QSU for *Tli*VMA intein (C1A), *Pho*VMA intein (C1A), and *Tli*VMA^ΔACD^ intein, respectively.

## References

[B1] Adams P. D. Grosse-Kunstleve R. W. Hung L.-W. Ioerger T. R. McCoy A. J. Moriarty N. W. (2002). PHENIX: Building New Software for Automated Crystallographic Structure Determination. Acta Crystallogr. D Biol. Cryst. 58 (11), 1948–1954. 10.1107/s0907444902016657 12393927

[B2] Aggarwal A. K. Rodgers D. W. Drottar M. Ptashne M. Harrison S. C. (1988). Recognition of a DNA Operator by the Repressor of Phage 434: a View at High Resolution. Science 242 (4880), 899–907. 10.1126/science.3187531 3187531

[B3] Anderson W. F. Ohlendorf D. H. Takeda Y. Matthews B. W. (1981). Structure of the Cro Repressor from Bacteriophage λ and its Interaction with DNA. Nature 290 (5809), 754–758. 10.1038/290754a0 6452580

[B54] Aranko A. S. Oeemig J. S. Kajander T. Iwaï H. (2013). Intermolecular Domain Swapping Induces Intein-Mediated Protein Alternative Splicing. Nat. Chem. Biol. 9 (10), 616–622. 10.1038/nchembio.1320 23974115

[B4] Aranko A. S. Wlodawer A. Iwaï H. (2014). Nature's Recipe for Splitting Inteins. Protein Eng. Des. Selection 27 (8), 263–271. 10.1093/protein/gzu028 PMC4133565 25096198

[B5] Argast G. M. Stephens K. M. Emond M. J. Monnat R. J. Jr (1998). I- Ppo I and I- Cre I Homing Site Sequence Degeneracy Determined by Random Mutagenesis and Sequential *In Vitro* Enrichment 1 1Edited by G. Smith. J. Mol. Biol. 280 (3), 345–353. 10.1006/jmbi.1998.1886 9665841

[B6] Arnold K. Bordoli L. Kopp J. Schwede T. (2006). The SWISS-MODEL Workspace: a Web-Based Environment for Protein Structure Homology Modelling. Bioinformatics 22 (2), 195–201. 10.1093/bioinformatics/bti770 16301204

[B7] Baek M. DiMaio F. Anishchenko I. Dauparas J. Ovchinnikov S. Lee G. R. (2021). Accurate Prediction of Protein Structures and Interactions Using a Three-Track Neural Network. Science 373 (6557), 871–876. 10.1126/science.abj8754 34282049 PMC7612213

[B8] Barzel A. Naor A. Privman E. Kupiec M. Gophna U. (2011). Homing Endonucleases Residing within Inteins: Evolutionary Puzzles Awaiting Genetic Solutions. Biochem. Soc. Trans. 39 (1), 169–173. 10.1042/bst0390169 21265767

[B9] Beyer H. M. Mikula K. M. Kudling T. V. Iwaï H. (2019). Crystal Structures of CDC21-1 Inteins from Hyperthermophilic Archaea Reveal the Selection Mechanism for the Highly Conserved Homing Endonuclease Insertion Site. Extremophiles 23 (6), 669–679. 10.1007/s00792-019-01117-4 31363851 PMC6801210

[B10] Beyer H. M. Mikula K. M. Li M. Wlodawer A. Iwaï H. (2020). The crystal Structure of the Naturally Split Gp41‐1 Intein Guides the Engineering of Orthogonal Split Inteins Fromcis‐splicing Inteins. FEBS J. 287 (9), 1886–1898. 10.1111/febs.15113 31665813 PMC7190452

[B11] Bowler M. W. Nurizzo D. Barrett R. Beteva A. Bodin M. Caserotto H. (2015). MASSIF-1: a Beamline Dedicated to the Fully Automatic Characterization and Data Collection from Crystals of Biological Macromolecules. J. Synchrotron Radiat. 22 (6), 1540–1547. 10.1107/s1600577515016604 26524320 PMC4629869

[B12] Brennan R. G. Matthews B. W. (1989). The helix-turn-helix DNA Binding Motif. J. Biol. Chem. 264 (4), 1903–1906. 10.1016/s0021-9258(18)94115-3 2644244

[B13] Burt A. Koufopanou V. (2004). Homing Endonuclease Genes: the Rise and Fall and Rise Again of a Selfish Element. Curr. Opin. Genet. Dev. 14 (6), 609–615. 10.1016/j.gde.2004.09.010 15531154

[B14] Carroll D. (2014). Genome Engineering with Targetable Nucleases. Annu. Rev. Biochem. 83, 409–439. 10.1146/annurev-biochem-060713-035418 24606144

[B15] Cermak T. Doyle E. L. Christian M. Wang L. Zhang Y. Schmidt C. (2011). Efficient Design and Assembly of Custom TALEN and Other TAL Effector-Based Constructs for DNA Targeting. Nucleic Acids Res. 39 (12), e82. 10.1093/nar/gkr218 21493687 PMC3130291

[B16] Chen V. B. Arendall W. B. Headd J. J. Keedy D. A. Immormino R. M. Kapral G. J. (2010). MolProbity: All-Atom Structure Validation for Macromolecular Crystallography. Acta Crystallogr. D Biol. Cryst. 66 (1), 12–21. 10.1107/s0907444909042073 20057044 PMC2803126

[B17] Chevalier B. S. Stoddard B. L. (2001). Homing Endonucleases: Structural and Functional Insight into the Catalysts of Intron/intein Mobility. Nucleic Acids Res. 29 (18), 3757–3774. 10.1093/nar/29.18.3757 11557808 PMC55915

[B18] Dalgaard J. Klar A. J. Moser M. J. Holley W. R. Chatterjee A. Mian I. S. (1997). Statistical Modeling and Analysis of the LAGLIDADG Family of Site- Specific Endonucleases and Identification of an Intein that Encodes a Site-specific Endonuclease of the HNH Family. Nucleic Acids Res. 25 (22), 4626–4638. 10.1093/nar/25.22.4626 9358175 PMC147097

[B19] Derbyshire V. Belfort M. (1998). Lightning Strikes Twice: Intron-Intein Coincidence. Proc. Natl. Acad. Sci. 95 (4), 1356–1357. 10.1073/pnas.95.4.1356 9465018 PMC33822

[B20] Derbyshire V. Wood D. W. Wu W. Dansereau J. T. Dalgaard J. Z. Belfort M. (1997). Genetic Definition of a Protein-Splicing Domain: Functional Mini-Inteins Support Structure Predictions and a Model for Intein Evolution. Proc. Natl. Acad. Sci. 94 (21), 11466–11471. 10.1073/pnas.94.21.11466 9326633 PMC23508

[B21] Dolinsky T. J. Nielsen J. E. McCammon J. A. Baker N. A. (2004). PDB2PQR: an Automated Pipeline for the Setup of Poisson-Boltzmann Electrostatics Calculations. Nucleic Acids Res. 32 (Web Server issue), W665–W667. 10.1093/nar/gkh381 15215472 PMC441519

[B22] Duan X. Gimble F. S. Quiocho F. A. (1997). Crystal Structure of PI-SceI, a Homing Endonuclease with Protein Splicing Activity. Cell 89 (4), 555–564. 10.1016/s0092-8674(00)80237-8 9160747

[B23] Dujon B. Beifort M. Butow R. A. Jacq C. Lemieux C. Perlman P. S. (1989). Mobile Introns: Definition of Terms and Recommended Nomenclature. Gene 82 (1), 115–118. 10.1016/0378-1119(89)90035-8 2555261

[B24] Emsley P. Lohkamp B. Scott W. G. Cowtan K. (2010). Features and Development of Coot. Acta Crystallogr. D Biol. Cryst. 66 (4), 486–501. 10.1107/s0907444910007493 20383002 PMC2852313

[B25] Eryilmaz E. Shah N. H. Muir T. W. Cowburn D. (2014). Structural and Dynamical Features of Inteins and Implications on Protein Splicing. J. Biol. Chem. 289 (21), 14506–14511. 10.1074/jbc.r113.540302 24695731 PMC4031508

[B26] Green C. M. Novikova O. Belfort M. (2018). The Dynamic Intein Landscape of Eukaryotes. Mobile DNA 9, 4. 10.1186/s13100-018-0111-x 29416568 PMC5784728

[B27] Grindl W. Wende W. Pingoud V. Pingoud A. (1998). The Protein Splicing Domain of the Homing Endonuclease PI-sceI Is Responsible for Specific DNA Binding. Nucleic Acids Res. 26 (8), 1857–1862. 10.1093/nar/26.8.1857 9518476 PMC147489

[B28] Guerrero F. Ciragan A. Iwaï H. (2015). Tandem SUMO Fusion Vectors for Improving Soluble Protein Expression and Purification. Protein Expr. Purif. 116, 42–49. 10.1016/j.pep.2015.08.019 26297996

[B29] Hiltunen M. K. Beyer H. M. Iwaï H. (2021). Mini-Intein Structures from Extremophiles Suggest a Strategy for Finding Novel Robust Inteins. Microorganisms 9 (6), 1226. 10.3390/microorganisms9061226 34198729 PMC8229266

[B30] Hirata R. Ohsumk Y. Nakano A. Kawasaki H. Suzuki K. Anraku Y. (1990). Molecular Structure of a Gene, VMA1, Encoding the Catalytic Subunit of H(+)-translocating Adenosine Triphosphatase from Vacuolar Membranes of *Saccharomyces cerevisiae* . J. Biol. Chem. 265 (12), 6726–6733. 10.1016/s0021-9258(19)39210-5 2139027

[B32] Ichiyanagi K. Ishino Y. Ariyoshi M. Komori K. Morikawa K. (2000). Crystal Structure of an Archaeal Intein-Encoded Homing Endonuclease PI-PfuI. J. Mol. Biol. 300 (4), 889–901. 10.1006/jmbi.2000.3873 10891276

[B33] Iwaï H. Mikula K. M. Oeemig J. S. Zhou D. Li M. Wlodawer A. (2017). Structural Basis for the Persistence of Homing Endonucleases in Transcription Factor IIB Inteins. J. Mol. Biol. 429 (24), 3942–3956. 10.1016/j.jmb.2017.10.016 29055778 PMC6309676

[B34] Jurrus E. Engel D. Star K. Monson K. Brandi J. Felberg L. E. (2018). Improvements to the APBS Biomolecular Solvation Software Suite. Protein Sci. 27 (1), 112–128. 10.1002/pro.3280 28836357 PMC5734301

[B35] Kabsch W. (2010). XDS. Acta Crystallogr. D Biol. Cryst. 66 (2), 125–132. 10.1107/s0907444909047337 20124692 PMC2815665

[B36] Langer G. Cohen S. X. Lamzin V. S. Perrakis A. (2008). Automated Macromolecular Model Building for X-ray Crystallography Using ARP/wARP Version 7. Nat. Protoc. 3 (7), 1171–1179. 10.1038/nprot.2008.91 18600222 PMC2582149

[B37] Liu X.-Q. (2000). Protein-splicing Intein: Genetic Mobility, Origin, and Evolution. Annu. Rev. Genet. 34, 61–76. 10.1146/annurev.genet.34.1.61 11092822

[B38] Maeder M. L. Thibodeau-Beganny S. Osiak A. Wright D. A. Anthony R. M. Eichtinger M. (2008). Rapid "Open-Source" Engineering of Customized Zinc-finger Nucleases for Highly Efficient Gene Modification. Mol. Cel 31 (2), 294–301. 10.1016/j.molcel.2008.06.016 PMC2535758 18657511

[B39] Matsumura H. Takahashi H. Inoue T. Yamamoto T. Hashimoto H. Nishioka M. (2006). Crystal Structure of Intein Homing Endonuclease II Encoded in DNA Polymerase Gene from Hyperthermophilic Archaeon Thermococcus Kodakaraensis Strain KOD1. Proteins 63 (3), 711–715. 10.1002/prot.20858 16493661

[B40] Moure C. M. Gimble F. S. Quiocho F. A. (2002). Crystal Structure of the Intein Homing Endonuclease PI-SceI Bound to its Recognition Sequence. Nat. Struct. Biol. 9 (10), 764–770. 10.1038/nsb840 12219083

[B41] Novikova O. Jayachandran P. Kelley D. S. Morton Z. Merwin S. Topilina N. I. (2016). Intein Clustering Suggests Functional Importance in Different Domains of Life. Mol. Biol. Evol. 33 (3), 783–799. 10.1093/molbev/msv271 26609079 PMC4760082

[B42] Panjikar S. Parthasarathy V. Lamzin V. S. Weiss M. S. Tucker P. A. (2005). Auto-Rickshaw: an Automated crystal Structure Determination Platform as an Efficient Tool for the Validation of an X-ray Diffraction experiment. Acta Crystallogr. D Biol. Cryst. 61 (4), 449–457. 10.1107/s0907444905001307 15805600

[B43] Pâques F. Duchateau P. (2007). Meganucleases and DNA Double-Strand Break-Induced Recombination: Perspectives for Gene Therapy. Curr. Gene Ther. 7 (1), 49–66. 10.2174/156652307779940216 17305528

[B44] Perler F. B. (1998). Protein Splicing of Inteins and Hedgehog Autoproteolysis: Structure, Function, and Evolution. Cell 92 (1), 1–4. 10.1016/s0092-8674(00)80892-2 9489693

[B45] Perler F. Olsen G. J. Adam E. (1997). Compilation and Analysis of Intein Sequences. Nucleic Acids Res. 25 (6), 1087–1093. 10.1093/nar/25.6.1087 9092614 PMC146560

[B46] Pettersen E. F. Goddard T. D. Huang C. C. Couch G. S. Greenblatt D. M. Meng E. C. (2004). UCSF Chimera?A Visualization System for Exploratory Research and Analysis. J. Comput. Chem. 25 (13), 1605–1612. 10.1002/jcc.20084 15264254

[B47] Pietrokovski S. (1994). Conserved Sequence Features of Inteins (Protein Introns) and Their Use in Identifying New Inteins and Related Proteins. Protein Sci. 3 (12), 2340–2350. 10.1002/pro.5560031218 7756989 PMC2142770

[B48] Sievers F. Wilm A. Dineen D. Gibson T. J. Karplus K. Li W. (2011). Fast, Scalable Generation of High‐quality Protein Multiple Sequence Alignments Using Clustal Omega. Mol. Syst. Biol. 7, 539. 10.1038/msb.2011.75 21988835 PMC3261699

[B49] Southworth M. W. Adam E. Panne D. Byer R. Kautz R. Perler F. B. (1998). Control of Protein Splicing by Intein Fragment Reassembly. EMBO J. 17 (4), 918–926. 10.1093/emboj/17.4.918 9463370 PMC1170441

[B50] Stoddard B. L. (2011). Homing Endonucleases: from Microbial Genetic Invaders to Reagents for Targeted DNA Modification. Structure 19 (1), 7–15. 10.1016/j.str.2010.12.003 21220111 PMC3038549

[B51] Swithers K. S. Senejani A. G. Fournier G. P. Gogarten J. P. (2009). Conservation of Intron and Intein Insertion Sites: Implications for Life Histories of Parasitic Genetic Elements. BMC Evol. Biol. 9, 303. 10.1186/1471-2148-9-303 20043855 PMC2814812

[B52] Taylor G. K. Petrucci L. H. Lambert A. R. Baxter S. K. Jarjour J. Stoddard B. L. (2012). LAHEDES: the LAGLIDADG Homing Endonuclease Database and Engineering Server. Nucleic Acids Res. 40 (Web Server issue), W110–W116. 10.1093/nar/gks365 22570419 PMC3394308

[B53] Volkmann G. Iwaï H. (2010). Protein Trans-splicing and its Use in Structural Biology: Opportunities and Limitations. Mol. Biosyst. 6 (11), 2110–2121. 10.1039/c0mb00034e 20820635

[B55] Wood D. W. Camarero J. A. (2014). Intein Applications: from Protein Purification and Labeling to Metabolic Control Methods. J. Biol. Chem. 289 (21), 14512–14519. 10.1074/jbc.r114.552653 24700459 PMC4031509

