# Supplementary material for: Structural Basis for the Propagation of Homing Endonuclease-Associated Inteins
Source: Front Mol Biosci. 2022 Mar 16;9:855511. doi: 10.3389/fmolb.2022.855511 (PMC8966425; doi:10.3389/fmolb.2022.855511)
Supplement: Supplementary file 1 [file DataSheet1.PDF]

Structural basis for the propagation of homing endonuclease-associated inteins

Hannes M. Beyer[1,2] and Hideo Iwaï[1,*]

[1]Institute of Biotechnology, University of Helsinki, Helsinki, FIN-00014, Finland
[2]Present: Institute of Synthetic Biology, Heinrich-Heine-University Düsseldorf, D-40225 Düsseldorf, Germany

Supporting Information

**Supplementary Materials and Methods**	Extended methods for crystallization, data collection, and structure determination.
**Supplementary Table S1**	Data collection and structure refinement of the three crystal structures of *Tli*VMA, *Tli*VMA$_{\Delta ACD}$, and *Pho*VMA inteins.
**Supplementary Table S2**	Plasmids, oligonucleotides, and linear dsDNA fragments used in this study.
**Supplementary Table S3**	Production of the recombinant proteins.
**Supplementary Table S4**	Pairwise comparison of the primary structures of the four inteins: *Pho*VMA, *Tli*VMA , *Pfu*VMA, and *Pab*VMA inteins.
**Supplementary Table S5**	DALI structural alignment of the ACD domain of *Tli*VMA intein against a PDB25 subset.
**Supplementary Figure S1**	Comparison of the structures corresponding to the HEN and HINT domains of *Tli*VMA *and Pho*VMA inteins, including functional cis-splicing assays.
**Supplementary Figure S2**	Determination of the cleavage sites by *Tli*VMA *and Pho*VMA inteins.
**Supplementary Figure S3**	Comparison of ACDs from the crystal structures of previously solved inteins.
**Supplementary Figure S4**	Cleavage specificities of *Tli*VMA and *Pho*VMA$_{Act}$ inteins and their temperature effects.
**Supplementary Figure S5**	Electrophoretic mobility shift assay of the isolated ACD of *Tli*VMA with two duplex DNAs containing the *Tli*VMA intein homing site.
**Supplementary Figure S6**	Sequential processing of homing and alternative sites by the *Pho*VMA$_{Act\text{-}ACD(Tli)}$ intein.
**Supplementary References**	

Supplementary Materials and Methods

Crystallization, data collection, and structure determination

Crystals were obtained at room temperature using the sitting drop vapour diffusion method. Diffracting crystals of *Pho*VMA intein were obtained by mixing 100 nL concentrated protein (29 mg/mL) with 100 nL mother liquor (100 mM HEPES, pH 8.0, 5 mM cadmium chloride, 5 mM magnesium chloride, 5 mM nickel (II) chloride, 10% (w/v) polyethylene glycol (PEG) 3350). For cryoprotection, 25% PEG 3550 was added to the drop. Data were collected at beamline i04 at Diamond Light Source (Didcot, UK) equipped with a Pilatus detector. Data were processed with XDS [1] at the nominal resolution of 2.49 Å (Table S1). The structure was solved by molecular replacement using Auto-Rickshaw [2] and ARP/wARP [3] for building initial partial models. A first solution was obtained using Auto-Rickshaw with a SWISSMODEL [4] generated from the intein homing endonuclease II of *Thermococcus kodakarensis* DNA polymerase as a search model (PDB: 2cw7). The model was further improved by several rounds of back and forth feeding into ARP/wARP and Auto-Rickshaw. A final model was generated with COOT [5] by the manual combination of fragments from several previously obtained Auto-Rickshaw and ARP/wARP models and manual building. The structure was refined using PHENIX [6] and COOT [5]. A total of 18 residues located in four loop regions were not modeled due to missing electron density information. The final model contains three Ni and two Cd metal ions.

*Tli*VMA intein crystals grew after mixing 100 nL concentrated protein (19.44 mg/mL) with 100 nL mother liquid (100 mM magnesium formate and 15% (w/v) PEG 3350). 25% PEG 3350 was added to the drop for cryoprotection. Data were recorded at beamline ID30A-1 (MASSIF-1) [7] at ESRF (Grenoble France) equipped with a Pilatus detector and were processed using XDS to 1.56 Å (Table S1). The structure was solved using the Auto-Rickshaw molecular replacement protocol with the *Pho*VMA intein (PDB: 7QST) as a search model. The obtained model was then used as a starting model for PHASER and rebuilt in place using PHENIX AutoBuild and COOT [5] and refined using PHENIX [6]. A stretch of sequence located in an unstructured loop region distant from endonuclease and intein active sites (residues 133-139) was built with ambiguity despite relatively low-density information. The remaining protein chain could be traced in the electron density without breaks (432 residues). Alternate conformations were modeled for residues Asn53, Arg55, Asn195, Arg263, Val336, Lys389, and Val399. The final model contains one formate molecule.

Diffracting crystals of *Tli*VMA$_{\Delta ACD}$ intein were obtained by mixing 100 nL concentrated protein (19.36 mg/mL) with 100 nL mother liquid (100 mM HEPES, pH 7.5, 70% (v/v) 2-methyl-2,4-pentanediol (MPD)). Data were collected at beamline i04 at Diamond Light Source (Didcot, UK) equipped with a Pilatus detector. Data were processed using XDS at the resolution of 1.90 Å (Table S1). The structure was solved by molecular replacement with PHASER [8] using the *Tli*VMA intein (PDB: 7QSS) as a search model and built and refined with COOT and PHENIX. The protein chain could be traced in the electron density without breaks except for the initial Ser residue (position -3) and a stretch of 7 residues (134-140) located in an unstructured region. The model includes one HEPES and one MPD molecule arising from the crystallization buffer.

Supplementary Table S1. Crystallographic data and statistics.

Intein	*Tli*VMA (C1A) intein	*Pho*VMA (C1A) intein	*Tli*$VMA_{\Delta ACD}$ (C1A, Δ333-339) intein
PDB ID code	7QSS	7QST	7QSU
Data collection			
Space group	$P2_12_12_1$	*C2*	$P2_1$
Unit cell			
a, *b*, *c* (Å);	48.80, 96.18, 96.78	91.18, 81.98, 67.73	69.6, 44.0,72.1
α, β, γ (°)	90, 90, 90	90, 112.84, 90	90, 104.6, 90
Wavelength (Å)	0.966	0.9795	0.9795
Resolution range (Å)	27.96 - 1.56 (1.65 - 1.56)	43.54 - 2.49 (2.64 - 2.49)	37.2 - 1.90 (2.01 - 1.90)
Total no. of reflections	285586	103947	223513
No. of unique reflections	65400 (10414)	16191 (2599)	33640 (5361)
Completeness, %	99.6 (99.5)	99.6 (99.4)	99.9 (99.8)
$< I / \sigma_I >$	13.32 (2.08)	17.28 (2.09)	19.30 (1.84)
R_{merge} (%)#	5.3 (59.6)	5.9 (71.4)	4.8 (85.8)
R_{meas} (%)†	6.1 (68.0)	6.4 (77.7)	5.2 (93.0)
$CC_{1/2}$&	0.999 (0.807)	0.999 (0.874)	1 (0.793)
Redundancy	4.4 (4.3)	6.4 (6.4)	6.6 (6.7)
Refinement			
Molecules / a.u.	1	1	1
Resolution range (Å)	27.96 - 1.56 (1.58 - 1.56)	43.51 - 2.49 (2.64 - 2.49)	37.2 - 1.90 (1.95 - 1.90)
No. of reflections (refinement / Rfree)	65394 / 3270	16178 / 810	33635 / 1682
R / R_{free} (%)‡	18.12 / 20.95	18.32 / 24.29	17.61 / 21.02
Number of atoms			
Protein	3493	2806	2926
Water	433	15	114
Ligand	3 (Formic acid)	5 (three Ni, two Cd)	23 (MPD,EPE)
R.m.s. deviations from ideal			
Bond lengths (Å)	0.010	0.009	0.008
Bond angles (°)	1.139	0.955	0.869
Ramachandran plot, %			
Favored (%)	96.3	96.3	98.1
Outliers (%)	0	0.3	0

The highest resolution shell is shown in parentheses.

#Rmerge = $\sum_h \sum_i | I_i - \langle I \rangle | / \sum_h \sum_i I_i$, where I_i is the observed intensity of the i-th measurement of reflection h, and $\langle I \rangle$ is the average intensity of that reflection obtained from multiple observations. &CC1/2 was defined in Karplus et al., 2012 [9]. †Defined in Diederichs & Karplus (1997) [10].

‡R = $\sum||Fo|-|Fc||/\sum|Fo|$, where Fo and Fc are the observed and calculated structure factors, respectively, calculated for all data. Rfree was defined in Brünger, 1992 [11].

Supplementary Table S2. Plasmids, oligonucleotides, and linear dsDNA fragments used in this study.

Genomic DNAs were purchased from the German collection of microorganisms and cell cultures (DSMZ) and American Type Culture Collection (ATCC). Abbreviations: ACD, Accessory domain; CBD, cellulose-binding domain; GB1, B1 domain of IgG binding protein G; H_6, hexahistidine tag; HEN, homing endonuclease; MBP, Maltose-binding protein; SUMO, small ubiquitin-like modifier; VMA, Vacuolar ATPase subunit A.

**Plasmid or duplex**	**Description**	**Reference**
Oligo-nucleotide duplex 1	Annealed duplex of oligonucleotides with the sequence 5’-GTAGCGGTAAAACAGT-AACACAGCACCAATTAGC and 5’-GGCTAATTGGTGCTGTGTTACTGTTTTACCG-CTA.	This work
Oligo-nucleotide duplex 2	Annealed duplex of oligonucleotides with the sequence 5’- GGGACCATTTGGTAGCG-GTAAAACAGTAACACAG and 5’-TGTGTTACTGTTTTACCGCTACCAAATGGTCCCC.	This work
pCARSF54	**P_{T7}::H_6-SUMO-*Pho*VMA(C1A) intein**<br>The sequence encoding *Pyrococcus horikoshii* VMA intein was PCR-amplified from genomic DNA (ATCC 700860D) using the oligonucleotides HK943: 5’-AGGATCCGG-TAAGGCCGTAGATGGAGATACTCTTG and HK944: 5’-GAGAAGCTTAGTTGTGAAG-CAATGTTGGCATG, thereby introducing the C1A substitution. The fragment was cloned into pHYRSF53-36 using *Bam*HI and *Hin*dIII.	This work
pCHRSF1	**P_{T7}::H_6-MBP-SUMO-*Tth*DnaE(C1A) intein**<br>Bacterial expression vector encoding the *Thermus thermophilus* DnaE intein with C1A substitution and N-terminal MBP, SUMO, and hexahistidine tags.	Unpublished
pHBRSF061	**P_{T7}::H_6-SUMO-*Pfu*VMA(C1A) intein**<br>The sequence encoding *Pyrococcus furiosus* VMA intein was PCR-amplified from genomic DNA (ATCC-43587D) using the oligonucleotides J616: 5’-TGTGTTGACGGG-GATACTTTAATTCTC and J617: 5’-GTTGTGAAGGAGTGTTGGCATGTTACC. The PCR product was further amplified using J606: 5’- GTGGATCCGGTAAAGCTGTTGAC-GGGGATACTTTAATTC and J607: 5’-CAGGTACCTTAGTTGTGAAGGAGTGTTGGC-ATGT, thereby introducing the C1A substitution. The PCR product was ligated into pHYRSF53 using the restriction enzymes *Bam*HI and *Kpn*I.	This work
pHBRSF062	**P_{T7}::H_6-SUMO-*Tli*VMA(C1A, N5D) intein**<br>The sequence encoding *Thermococcus litoralis* VMA intein was PCR-amplified from genomic DNA (DSM-5473) using the oligonucleotides J606: 5’-GTGGATCCGGTAAAG-CTGTTGACGGGGATACTTTAATTC and J617: 5’-GTTGTGAAGGAGTGTTGGCATGT-TACC followed by a second amplification with J606 and J607: 5’-CAGGTACCTTAGTT-GTGAAGGAGTGTTGGCATGT. The product carrying C1A and N5D substitutions was ligated into pHYRSF53 using *Bam*HI and *Kpn*I.	This work
pHBRSF063	**P_{T7}::H_6-MBP-SUMO-*Tli*VMA(C1A) intein**<br>The sequence encoding *Thermococcus litoralis* VMA intein was PCR-amplified from pHBRSF62 using the oligonucleotides HB084: 5’-GTGGATCCGGTAAAGCTGTTGAC-GGGAATAC and HB029: 5’-GCGGCCGCAAGCTTAGTTGTGCACGGCGAACC. The product was ligated into pCHRSF1 using *Bam*HI and *Not*I.	This work

Plasmid or duplex	Description	Reference
pHBRSF064	**P_{T7}::H_6-MBP-SUMO-*Pfu*VMA(C1A) intein**<br>The *Pfu*VMA(C1A) intein was transferred from pHBRSF061 to pCHRSF1 by digestion and ligation using the restriction enzymes BamHI and NotI, thereby adding MBP to the N-terminal fusion.	This work
pHBRSF065	**P_{T7}::H_6-MBP-SUMO-*Pab*VMA(C1A) intein**<br>The sequence encoding *Pyrococcus abyssi* VMA intein was PCR-amplified from genomic DNA (DSM-25543) using the oligonucleotides HB085: 5'-TTGGATCCGGGAA-GGCTGTTGATGGGGATACACTAG and HB011: 5'-GAGTGCGGCCGCTTAGTTGTG-AAGGAGCGTTGGCATG and cloned into pCHRSF1 using the restriction enzymes *Bam*HI and *Not*I.	This work
pHBRSF066	**P_{T7}::H_6-SUMO-*Pho*VMA(C1A, N159D) intein**<br>Plasmid pCARSF54 was PCR-amplified using oligonucleotides HB086: 5'-GATATCTA-ATGGCCGATGGAACCTTGAAGTCTGGGATC and HB087: 5'-TCCATCGGCCATTAG-ATATCCTAGGAATTCCG. The PCR product was ligated using Gibson cloning resulting in the N159D substitution.	This work
pHBRSF067	**P_{T7}::H_6-SUMO-*Pho*VMA (C1A, N159D, G252CDGYYHER) intein**<br>Plasmid pHBRSF066 was PCR-amplified using oligonucleotides HB088: 5'-TGCGATG-GCTATTATCATGAACGCAAAGAGGAAGTTGAAATAACACTGG and HB089: 5'-GCG-TTCATGATAATAGCCATCGCATTTAATATAGGCATTTACAAATGCCCTAAC. The PCR product was ligated using Gibson cloning resulting in the substitution of G252 with the amino acid sequence 'CDGYYHER'.	This work
pHBDuet068	**P_{T7}:: H_6-GB1-*Tli*VMA-GB1**<br>Plasmid pHBRSF63 was PCR-amplified using oligonucleotides HB094: 5'- AGGATCC-GGTAAATGTGTTGACGGGAATACTTTAGTGCTTAC and J602: 5'-TTGGTACCAGTC-ACCGTGTTGTGAAGCAATGTTGGCATG. The PCR product was ligated into the *Bam*HI/*Kpn*I sites of pSKDuet16 [12] to test *cis*-splicing activity using two GB1s.	This work
pHBDuet072	**P_{T7}:: H_6-GB1-*Tli*VMA(delHEN123-388)-GB1**<br>Plasmid pHBDuet072 was created from pHBDuet068 by inverse PCR using the oligonucleotides J704: 5'-GTCGTGAAGAAAAATGGAAAGCACATTCTTTTTGATGAAG and J705: 5'-AAAGAATGTGCTTTCCATTTTTCTTCACGACAGCTATTC.	This work
pHBRSF075	**P_{T7}::H_6-MBP-SUMO-*Tli*VMA(C1A, ΔACD 333-390) intein**<br>Plasmid pHBRSF063 was PCR-amplified using oligonucleotides HB098: 5'-AACATTG-ACATAGTTATTCTTTTTGATGAAGTAGTTGAAG and HB099: 5'-TTCATCAAAAAGA-ATAACTATGTCAATGTTCGTGTACC. The PCR product was circularized by using Gibson cloning, resulting in deletion of the accessory domain (residues 333-390), leaving some linker sequence.	This work
pHBRSF079	**P_{T7}::H_6-SUMO-*Pho*VMA (C1A, N159D, G252CDGYYHER, *Tli*VMA ACD domain) intein**<br>The sequence encoding the accessory domain of *Tli*VMA intein was PCR-amplified from pHBRSF063 using the oligonucleotides HB115: 5'-AGTAGAAGGGGCCCAGTTG-AGGTTGAAAGTATATACAATG and HB116: 5'-GTCAAAGATGATGTGCTTGAGGTG-ATTCTCGG. To replace the original accessory domain of activated *Pho*VMA intein, pHBRSF067 was PCR-amplified using the oligonucleotides HB113: 5'-CACCTCAAGC-	This work

Plasmid or duplex	Description	Reference
	ACATCATCTTTGACGAGGTCATTGAC and HB114: 5'-AACCTCAACTGGCCCCTT-CTACTTACCCTAACC. Both PCR products were joined using Gibson cloning.	
pHBDuet080	**P_{T7}:: H_6-GB1-*Pho*VMA(delHEN123-388)-GB1**<br>Plasmid pHBDuet080 was derived from pLKDuet24 by inverse PCR using oligonucleotides HB117: 5'-CGTAAAGAAAAATGGACAGCACATCATCTTTGACGAG and HB118:: 5'-TGATGTGCTGTCCATTTTTCTTTACGACTGCTATTCTATCTC.	This work
pHBRSF082	**P_{T7}::H_6-SUMO-*Tli*VMA ACD domain (337-393)**<br>The ACD domain of *Tli*VMA (res 337-393) was PCR-amplified from pHBRSF063 using the oligonucleotides L60: 5'-GAACAGATTGGTGGATCCGTTGAGGTTGAAAGTATAT-AC and L61: 5'-TGCGGCCGCAAGCTTAGTGCTTGAGGTGATTCTCGG. pHYRSF53 was amplified using J502: 5'-TAAGCTTGCGGCCGCACTC and HB030: 5'-GGATCCA-CCAATCTGTTCTCTG. The fragments were joined by Gibson cloning.	This work
pHBRSF083	**P_{T7}::H_6-MBP-SUMO-*Tli*VMA(C1A, *Pfu*VMA ACD domain) intein**<br>To replace the accessory domain of the *Tli*VMA intein with the ACD from *Pyrococcus furiosus*, the *Pfu*VMA ACD was PCR-amplified from pHBRSF064 using the oligonucleotides L75:5'-GAACATTGACATAGTTCCAGTTGACGTTGAGAGTATATAC and L76: 5'-CTTCATCAAAAAGAATGTGCTGAAGATGGTTCTCTGCT. The gene fragment was inserted into the *Tli*VMA intein coding sequence by amplification of pHBRSF063 using L80: 5'-CACATTCTTTTTGATGAAGTAGTTG and L79: 5'-TCAACT-GGAACTATGTCAATGTTCG followed by Gibson assembly of the two fragments.	This work
pHBRSF084	**P_{T7}::H_6-MBP-SUMO-*Tli*VMA(C1A, *Pab*VMA ACD domain) intein**<br>To replace the accessory domain of the *Tli*VMA intein with the ACD from *Pyrococcus abyssi*, the *Pab*VMA ACD was PCR-amplified from pHBRSF065 using the oligonucleotides L77: 5'-GAACATTGACATAGTTCCAGTGGAGGTTGAGGAGCTA and L78: 5'- CTTCATCAAAAAGAATGTGAGTTAAGTGGTTCTCAGCTA. The gene fragment was inserted into the *Tli*VMA intein coding sequence as described for pHBRSF083.	This work
pHBRSF161	**P_{T7}::H_6-MBP-SUMO-*Tli*VMA(C1A, 434-repressor domain) intein**<br>The 434-phage repressor domain was PCR-amplified from pHYBAD61 using the oligo-nucleotides HB121: 5'-GACATAGTTCCAGTTGAGGGTAGTATTTCTTCCAGGGTAAA-AAGC and HB122: 5'-CTTCATCAAAAAGAATGTGACCGGTGCCATTGAGCAGCC and inserted into the *Tli*VMA intein coding sequence as described for pHBRSF083.	This work
pHYBAD61	**P_{BAD}::*Ssp*DnaE_C intein-*434-repressor***<br>Bacterial expression vector encoding the C-terminal split fragment of the *Synechocystis species* (*Ssp)* DnaE intein fused to the 434-bacteriophage repressor domain.	Unbublished
pHYRSF53-36	**P_{T7}::H_6-SUMO-*Npu*DnaE_{N123} intein**<br>Bacterial expression vector encoding the N-terminal fragment of the split DnaE intein of *Nostoc punctiforme* with an N-terminal H_6-SUMO fusion.	[13]
pHYRSF53	**P_{T7}::H_6-SUMO-*Npu*DnaE_{N102} intein-CBD**<br>Bacterial expression vector encoding hexahistidine-tagged fusion protein of SUMO, N-terminal 102 residues of the *Nostoc punctiforme* DnaE intein, and CBD.	Addgene #64696

**Plasmid or duplex**	**Description**	**Reference**
pLKRDuet28	**P_{T7}:: H_6-GB1-*Pho*VMA-GB1**<br>Plasmid pCARSF54 was PCR-amplified using oligonucleotides J601:5'-AGGATCCGGTAAGTGCGTAGATGGAGATACTCTT and J602: 5'-TTGGTACCAGTC-ACCGTGTTGTGAAGCAATGTTGGCATG. The PCR product was ligated into *Bam*HI/*Kpn*I sites of pSKDuet16 [12] for testing *cis*-splicing with two GB1s as the exteins.	This work
*Pho*VMA<br>750 bp substrate	The 750 bp linear DNA fragment containing *Pho*VMA intein homing site was generated by assembly PCR from genomic DNA (ATCC 700860D) using the oligonucleotides HB073: 5'-AGAGATCGTTGAGATTGCGG and HB074: 5'-ATGCTGAGTCACCGTCTT-ACCGCTGCCGAAAGG, and HB075: 5'-TTCGGCAGCGGTAAGACGGTGACTCAGC-ATCAGC and HB076: 5'-GTGTTCTGAACTACAGGTTCACTG.	This work
*Tli*VMA<br>750 bp substrate	The 750 bp linear DNA fragment containing *Tli*VMA intein homing site was generated by assembly PCR from genomic DNA (DSM-5473) using the oligonucleotides HB090: 5'-CGAGATATTAGAGATTGCTGAAGAGG and HB091: 5'-GTGCTGTGTTACTGTTTT-ACCGCTACCAAATGGTCCC, and HB092: 5'-TTTGGTAGCGGTAAAACAGTAACACA-GCACCAATTAGC and HB093: 5'-GTGTTCTGTACGACAGGATCG.	This work
*Pho*VMA<br>2037 bp substrate	The 2037 bp linear DNA fragment containing only the *Tli*VMA intein alternative homing site was generated by PCR from genomic DNA (DSM-5473) using the oligonucleotides HB090: 5'-CGAGATATTAGAGATTGCTGAAGAGG and HB093: 5'-GTGTTCTGTACG-ACAGGATCG.	This work

Supplementary Table S3. Production of the recombinant proteins. All proteins were produced in *E. coli* T7 Express strain using LB medium supplemented with 25 µg/mL kanamycin and were induced at the given OD_{600} with 1 mM IPTG. 2 L cultures originating from a single colony were precultured overnight at 30°C, 250 rpm and were diluted 50x for inoculation of the expression culture. Abbreviations: IPTG, isopropyl-β-D-thiogalactoside; LB, lysogeny broth. For further abbreviations see Supporting Table S2.

**Vector**	**Protein**	**Temp**	**Medium**	**Culture volume**	**Density**	**Duration**
pCARSF54	H_6-SUMO-*Pho*VMA(C1A) intein	37°C	LB	2 L	OD_{600} = 0.6	4 h
pHBRSF063	H_6-MBP-SUMO-*Tli*VMA(C1A) intein	18°C	LB	2 L	OD_{600} = 0.6	20 h
pHBRSF067	H_6-SUMO-*Pho*VMA_{Act} (C1A, N159D, G252CDGYYHER) intein	37°C	LB	2 L	OD_{600} = 0.6	4 h
pHBRSF075	H_6-MBP-SUMO-*Tli*$VMA_{\Delta ACD}$ (C1A, ΔACD 333-390) intein	18°C	LB	2 L	OD_{600} = 0.6	20 h
pHBRSF079	H_6-SUMO-*Pho*$VMA_{Act\text{-}ACD(\textit{Tli})}$ (C1A, N159D, G252CDGYYHER, *Tli*VMA ACD domain) intein	37°C	LB	2 L	OD_{600} = 0.6	4 h
pHBRSF082	H_6-SUMO-*Tli*VMA ACD domain (337-393)	37°C	LB	1 L	OD_{600} = 0.6	3 h
pHBRSF083	H_6-MBP-SUMO-*Tli*VMA(C1A, *Pfu*VMA ACD domain) intein	18°C	LB	2 L	OD_{600} = 0.6	20 h
pHBRSF084	H_6-MBP-SUMO-*Tli*VMA(C1A, *Pab*VMA ACD domain) intein	18°C	LB	2 L	OD_{600} = 0.6	20 h
pHBRSF161*	H_6-MBP-SUMO-*Tli*VMA(C1A, 434-repressor domain) intein	18°C	LB	2 L	OD_{600} = 0.54	20 h

* Plasmid was co-transformed with pRARE to account for rare codons and cultures contained 5 µg/mL chloramphenicol.

Supplementary Table S4. Pairwise intein amino-acid sequence alignment identity matrix. Values are given in percent identity. The matrix was created with Clustal2.1. Abbreviations: *Pab*, *Pyrococcus abyssi*; *Pfu*, *Pyrococcus furiosus*; *Pho*, *Pyrococcus horikoshii*; *Tli*, *Thermococcus litoralis*; VMA, subunit A of the Vacuolar-type ATP synthase.

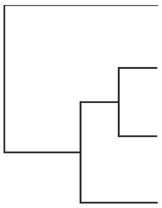

Intein:	*Pho*VMA	*Tli*VMA	*Pfu*VMA	*Pab*VMA
*Pho*VMA	**100.00**	73.74	73.74	65.25
*Tli*VMA	73.74	**100.00**	85.68	75.35
*Pfu*VMA	73.74	85.68	**100.00**	77.93
*Pab*VMA	65.25	75.35	77.93	**100.00**

Supplementary Table S5: Results from the DALI server for the structural alignment of the ACD of (residues 330 - 390) of *Tli*VMA inteins against a PDB25 subset [14].

Chain	Z	R. M. S. D.	ID (%)	Description
6h4c-D	4.08	3.00	19	DUTPASE
1utx-A	4.04	2.05	16	CYLR2
6jq1-B	4.04	3.01	15	TRANSCRIPTIONAL REGULATOR, XRE FAMILY
3omt-A	4.03	2.05	14	UNCHARACTERIZED PROTEIN
3eus-A	4.02	3.01	7	DNA-BINDING PROTEIN
3f51-A	4.02	2.07	14	CLP GENE REGULATOR (CLGR)
5w4m-B	4.02	2.06	10	TRANSCRIPTIONAL REGULATOR
5fd4-A	4.02	3.01	11	COMR
2grm-A	4.01	2.05	14	PRGX
4ryk-A	4.01	2.05	16	LMO0325 PROTEIN
5v5t-A	4.00	3.04	9	CONSERVED DOMAIN PROTEIN
1adr-A	4.00	3.02	10	**P22 C2 REPRESSOR**
2qfc-A	4.00	2.07	6	PLCR PROTEIN
4yba-B	4.00	3.01	9	REGULATORY PROTEIN C
1b0n-A	3.09	2.07	8	PROTEIN (SINR PROTEIN)
6af3-D	3.09	2.06	15	HIGB TOXIN
5d50-A	3.09	2.05	12	REPRESSOR;
3ivp-A	3.09	3.04	11	PUTATIVE TRANSPOSON-RELATED DNA-BINDING PROTEIN
3kxa-A	3.08	2.03	20	PUTATIVE UNCHARACTERIZED PROTEIN
5woq-B	3.07	2.07	8	TRANSCRIPTIONAL REGULATOR CLGR
4i6u-A	3.07	2.02	16	REGULATORY PROTEIN
2ef8-A	3.07	3.02	12	PUTATIVE TRANSCRIPTION FACTOR
4ghj-B	3.07	2.07	12	PROBABLE TRANSCRIPTIONAL REGULATOR
2ewt-A	3.06	3.00	16	PUTATIVE DNA-BINDING PROTEIN
3op9-A	3.06	3.00	12	PLI0006 PROTEIN
4j1x-C	3.05	2.05	17	EPOXIDASE
4o8b-A	3.05	2.04	10	UNCHARACTERIZED PROTEIN
5muu-D	3.05	2.06	15	MAJOR INNER PROTEIN P1
1zug-A	3.04	2.09	14	**PHAGE 434 CRO PROTEIN**
3bd1-A	3.04	2.09	15	CRO PROTEIN
4yg1-A	3.04	2.06	26	ANTITOXIN HIPB
2a6c-A	3.03	2.09	13	HELIX-TURN-HELIX MOTIF
2o38-B	3.03	2.04	8	HYPOTHETICAL PROTEIN
3b7h-A	3.03	2.08	16	PROPHAGE LP1 PROTEIN 11
2ofy-A	3.02	2.08	12	PUTATIVE XRE-FAMILY TRANSCRIPTIONAL REGULATOR
3qf3-A	3.02	3.05	10	ESX-1 SECRETION-ASSOCIATED REGULATOR ESPR
3pxp-A	3.02	2.09	13	HELIX-TURN-HELIX DOMAIN PROTEIN
4yar-A	3.02	2.08	14	2-HYDROXYETHYLPHOSPHONATE DIOXYGENASE
3bdn-A	3.01	3.00	8	LAMBDA REPRESSOR
6u0i-A	3.01	3.00	18	ANTITOXIN HICB
3vk0-A	3.00	2.09	14	TRANSCRIPTIONAL REGULATOR
2ppx-A	3.00	2.03	9	UNCHARACTERIZED PROTEIN ATU1735
2auw-B	2.09	2.04	6	HYPOTHETICAL PROTEIN NE0471
3mlf-A	2.09	3.01	10	TRANSCRIPTIONAL REGULATOR
2jvl-A	2.09	2.09	15	TRMBF1
2eby-A	2.08	2.07	13	PUTATIVE HTH-TYPE TRANSCRIPTIONAL REGULATOR YBAQ
1dq3-A	2.08	2.09	8	ENDONUCLEASE
3j16-B	2.08	3.03	10	DOM34P
3gn5-A	2.08	2.09	12	HTH-TYPE TRANSCRIPTIONAL REGULATOR MQSA (YGIT/B30)
2kpj-A	2.08	2.09	10	SOS-RESPONSE TRANSCRIPTIONAL REPRESSOR, LEXA

Supplementary Figures

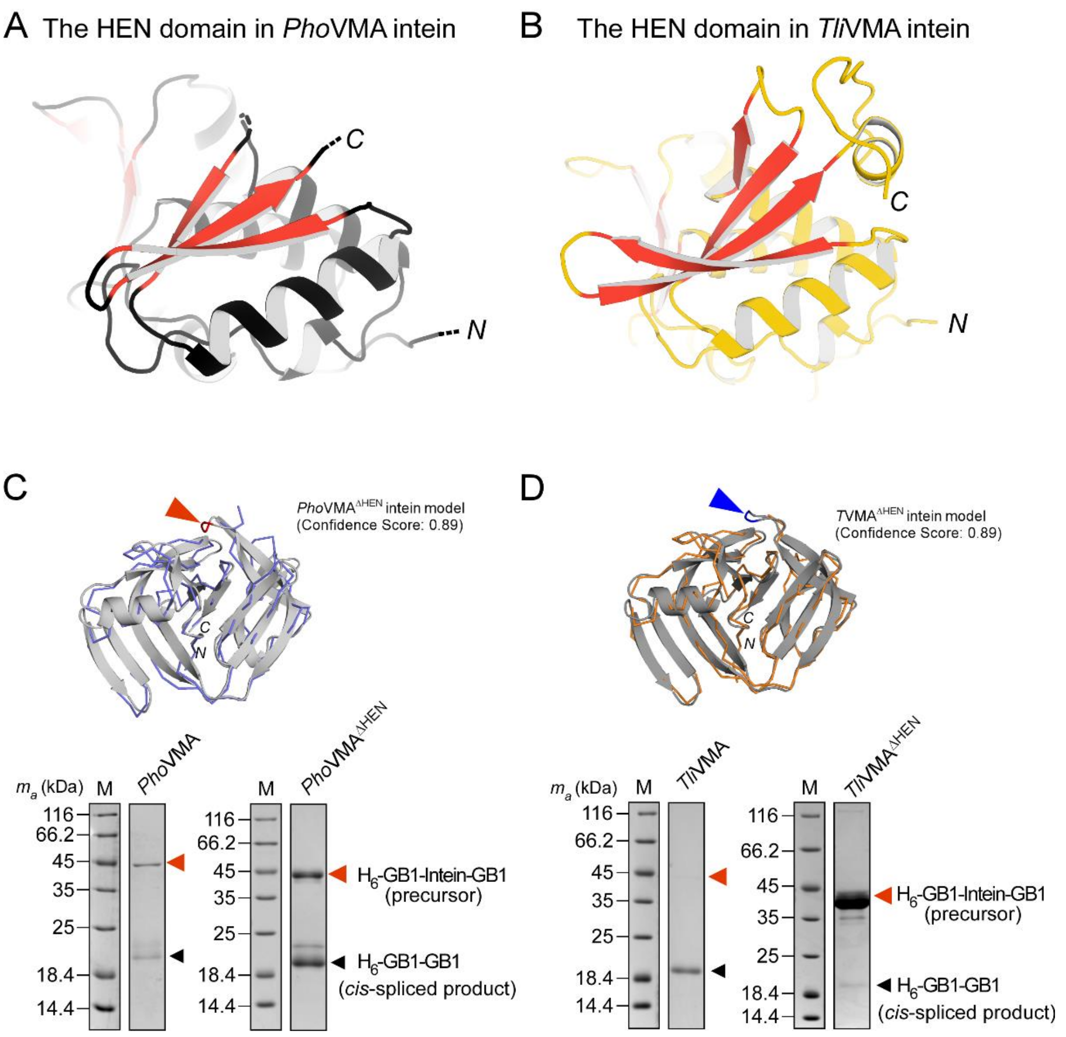

Supplementary Figure S1. Comparison of the HEN domains of *Pho*VMA (**A**) and *Tli*VMA (**B**) inteins. Regions corresponding to shortened sheets in the HEN domain from *Pho*VMA intein are highlighted in red. *N* and *C* denote the termini. (**C**) A structural model of *Pho*VMA$^{\Delta HEN}$ intein superimposed with the HINT domain of *Pho*VMA intein (in light blue). The "NG" sequence is colored in red. SDS-PAGE analysis of *Cis*-splicing of *Pho*VMA and *Pho*VMA$^{\Delta HEN}$ inteins. (**D**) A structural model of *Tli*VMA$^{\Delta HEN}$ intein superimposed with the HINT domain of *Tli*VMA intein (in orange). The "NG" sequence is colored in blue. SDS-PAGE analysis of *cis*-splicing of *Tli*VMA and *Tli*VMA$^{\Delta HEN}$ inteins.

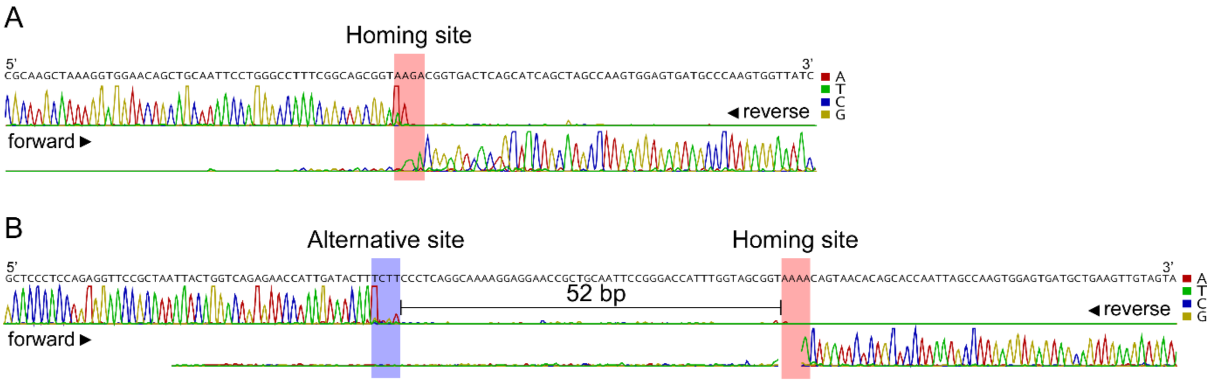

C

```
TliVMA      CGAGATATTAGAGATTGCTGAAGAGGGAGATTACACGATAGAAGAAGTCATTGCAAAGGT   60
PhoVMA      AGAGATCGTTGAGATTGCGGAGGAAGGAGACTACACAATAGAAGAGGTTATAGCAAAGGT   60
             *****  * ******** ** ** ***** ***** ******** ** ** ********

TliVMA      TAAAACCCCAGACGGGGAGATAAAGGAGCTTAAGATGTACCAAAGATGGCCCGTTAGAGT   120
PhoVMA      AAAGACTCCTAGCGGTGAGATAAAAGAGCTTAAGATGTATCAGAGGTGGCCCGTTAGAGT   120
             ** ** **   *** ******** ************** ** ** **************

TliVMA      TAAAAGACCATACAAAGAGAAGCTCCCTCCAGAGGTTCCGCTAATTACTGGTCAGAGAAC   180
PhoVMA      GAAGAGGCCTTACAAGGAGAAGCTTCCACCTGAAGTTCCTCTAATAACTGGACAGAGGGT   180
             ** ** ** ***** ******** ** ** ** ***** ***** ***** *****

TliVMA      CATTGATACTTTCTTCCCTCAGGCAAAAGGAGGAACCGCTGCAATTCCGGGACCATTTGG   240
PhoVMA      AATAGATACCTTCTTCCCGCAAGCTAAAGGTGGAACAGCTGCAATTCCTGGGCCTTTCGG   240
             ** ***** ******** ** ** ***** ***** *********** ** ** ** **

TliVMA      TAGCGGTAAAACAGTAACACAGCACCAATTAGCCAAGTGGAGTGATGCTGAAGTTGTAGT   300
PhoVMA      CAGCGGTAAGACGGTGACTCAGCATCAGCTAGCCAAGTGGAGTGATGCCCAAGTGGTTAT   300
             ******** ** ** ** ***** **  *******************  **** **  *

TliVMA      ATACATTGGTTGTGGCGAGAGAGGAAACGAGATGACGGATGTTCTTGAAGAGTTCCCCAA   360
PhoVMA      CTATATTGGTTGTGGAGAGAGAGGAAACGAGATGACGGATGTTCTTGAAGAGTTCCCCAA   360
             ** *********** ********************************************

TliVMA      GCTGAAAGACCCGAGAACAGGCAAACCATTGATGGAGAGAACCGTTCTCATAGCAAACAC   420
PhoVMA      GCTTAAGGATCCTAAGACTGGTAAGCCATTAATGGAAAGAACCGTTTTAATAGCTAACAC   420
            *** ** ** ** *  ** ** ** ***** ***** ********* * ***** *****

TliVMA      CTCAAACATGCCCGTTGCTGCTAGAGAAGCCTCTATTTATACGGGAATTACAATAGCAGA   480
PhoVMA      CTCTAACATGCCTGTGGCCGCTAGAGAAGCCTCTATTTACACCGGAATCACGATAGCCGA   480
            *** ******** ** ** ******************** ** ***** ** ***** **

TliVMA      GTATTTCAGAGATATGGGTTATAACGTAGCTTTAATGGCAGATTCAACTTCAAGATGGGC   540
PhoVMA      GTACTTCAGGGATATGGGATATGATGTAGCCCTAATGGCTGATTCAACCTCAAGATGGGC   540
            *** ***** ******** *** * *****  ******* ******** ***********

TliVMA      CGAAGCTTTGAGAGAAATCTCAGGAAGACTTGAGGAAATGCCCGGTGAAGAAGGTTATCC   600
PhoVMA      CGAAGCCCTGAGAGAGATCTCAGGTAGACTTGAGGAAATGCCTGGTGAAGAGGGATATCC   600
            ******  ******* ******** ***************** ******** ** *****

TliVMA      AGCTTACCTAGCTTCAAAGATAGCCGAATTCTATGAAAGAGCCGGAAGGGTTAGAACGTT   660
PhoVMA      AGCTTACTTAGCCTCAAAGCTTGCCGAATTCTACGAGAGAGCAGGTAGAGTTGTTACCCT   660
            ******* **** ****** * *********** ** ***** ** ** ***   **  *

TliVMA      AGGAAGTGACGGTAGAATTGGAAGTGTAAGCGTTATTGGAGCTGTTTCTCCGCCAGGTGG   720
PhoVMA      TGGAAGTGACTACAGGGTTGGAAGCGTTTCTGTAATAGGAGCGGTGTCACCTCCTGGAGG   720
             *********   **  ******* **    ** ** ***** ** ** ** ** ** **

TliVMA      TGATTTGAGCGATCCTGTCGTACAGAACAC   750
PhoVMA      AGACTTCAGTGAACCTGTAGTTCAGAACAC   750
             ** ** ** ** ***** ** ********
```

Supplementary Figure S2. Determination of the cleavage sites by *Tli*VMA *and Pho*VMA inteins. (**A**) The Sanger sequencing chromatograph from the digested 750-bp *Pho* DNA substrate, which was digested overnight with *Pho*VMA$_{Act}$ intein. The cleavage products were sequenced, revealing the central four base pairs where cleavage occurs (5'-AAGA-3', red). (**B**) The Sanger sequencing chromatograph from the digested 750-bp DNA*Tli* substrate, which was overnight digested with *Tli*VMA intein. The 500 and ~200 bp cleavage products were sequenced, revealing an alternative site (5'-AAGA-3', blue) cleavage in addition to the expected homing site (5'-AAAA-3', red), separated by 52 bp. In (A)-(B)**,** Note that *Taq* polymerase commonly used in Sanger sequencing possesses non-template adenylation activity resulting in significant A peaks (T peaks in the reverse reading), indicating termination of the template. (**C**) The sequence alignment of the 750 bp *Tli* and *Pho* DNA. The homing and alternative sites are highlighted in red and blue, respectively.

A

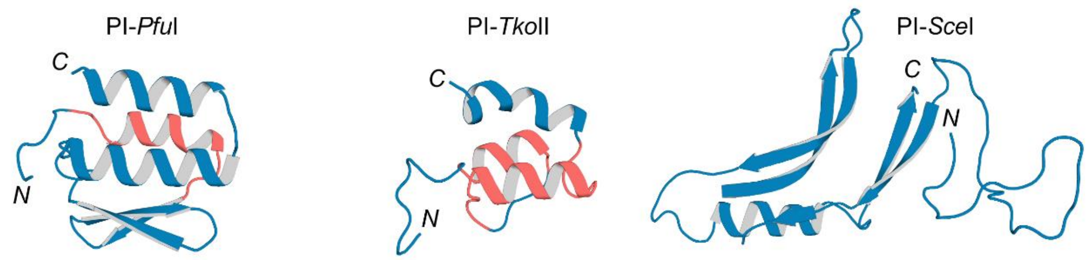

B

```
TliVMA   ACD  DIVPVEVESIYNALGRPYSELKGEGIEIHNYLNGENMTYETFRKFAKLVGLEEVAENHL-
PI-PfuI  ACD  -----------GN-------------------FGLPLNFNAFKEWASEYGVEFKTNGSQT
                         .                     *  :.:::*:::*.  *:*  ::.

TliVMA   ACD  -----------------------------------------KH--------
PI-PfuI  ACD  IAIINDERISLGQWHTRNRVSKAVLVKMLRKLYEATKDEEVKRMLHLIEGL
                                                       *:
```

C

```
TliVMA    ACD  -------DIVPVEVESIYNALGRPYSELKGEGIEIHNYLNGENMTYETFRKFAKLVGLEE
PI-TkoII  ACD  KKNAYYSHVIPKEVLS--EVFGKVF---------------QKNVSPQTFRKMVEDGRLDP
                      .::* ** *  :.:*: :                :*:: :****:.:   *:

TliVMA    ACD  VAENHLKH----
PI-TkoII  ACD  EKAQRLSWLIEG
                  ::*.
```

Supplementary Figure S3. Comparison of ACDs from the crystal structures of previously solved inteins. (**A**) The structures of the corresponding ACD domains from PI-*Pfu*I (*Pyrococcus furiosus*, PDB: 1dq3), PI-*Tko*II (*Thermococcus kodakaraensis*, PDB: 2cw7), and PI-*Sce*I (Saccharomyces cerevisiae, PDB: 1lws). The sequence with partial similarity to the ACD of the *Tli*VMA intein is colored in red (see B and C). (**B**) A sequence alignment of the ACDs of the *Tli*VMA and PI-*Pfu*I inteins. (**C**) A sequence alignment of the ACDs of *Tli*VMA and PI-*Tko*II inteins. The sequence with partial similarity is highlighted in red**.**

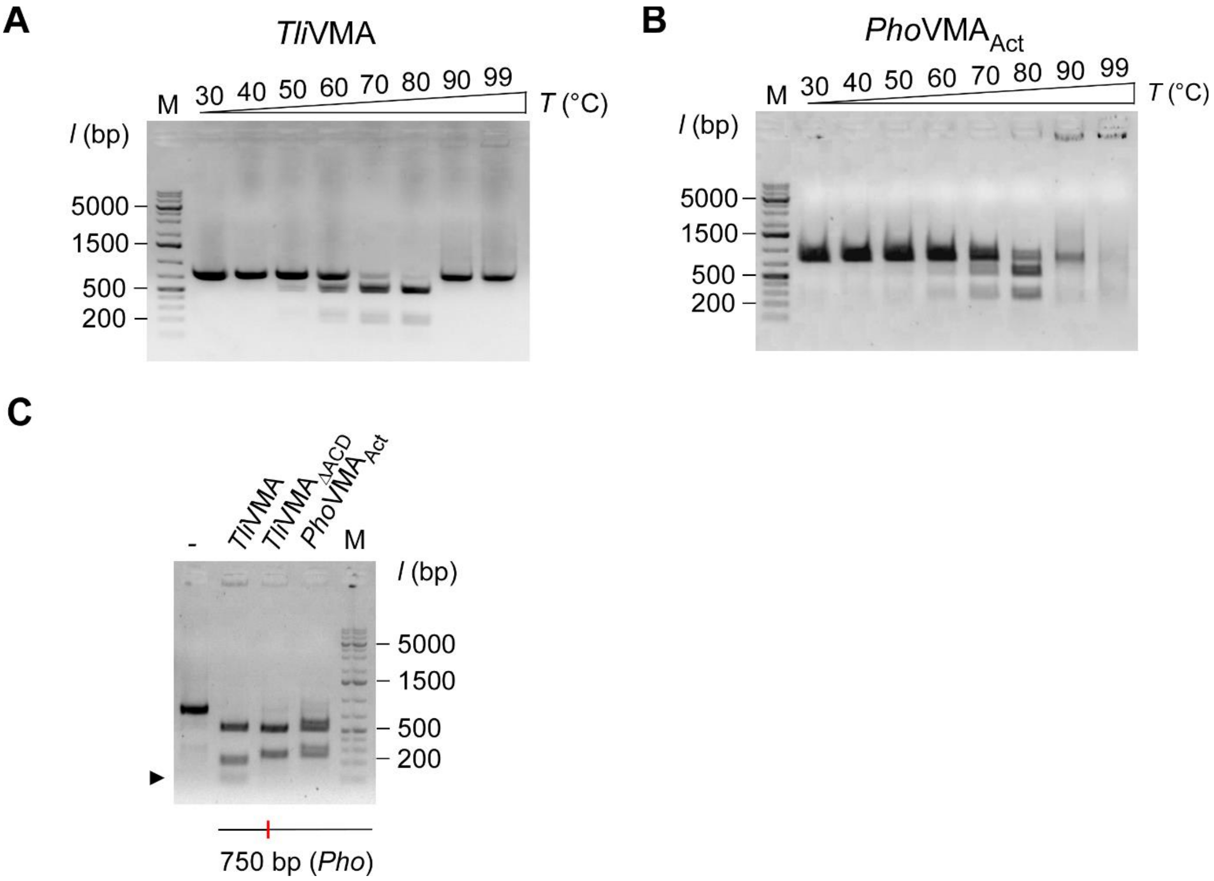

Supplementary Figure S4. Cleavage specificity of the *Tli*VMA and *Pho*VMA$_{Act}$ inteins and their temperature effects. (**A**) Temperature effect on DNA cleavage by the *Tli*VMA intein upon incubation with 750 bp *Tli* substrate DNA for 2 h at the indicated temperatures. (**B**) Temperature effect on DNA cleavage by the *Pho*VMA$_{Act}$ intein upon incubation with 750 bp *Pho* substrate DNA for 2 h at the indicated temperatures. (**C**) Experiment as in Fig. 4C but using 750 bp substrate DNA generated from *Pho* genomic DNA instead. The arrowhead indicates product P3.

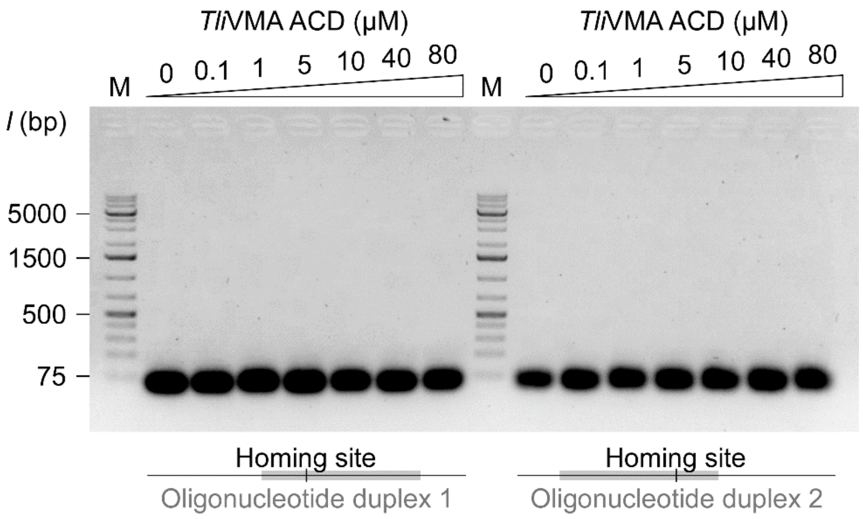

Supplementary Figure S5. Electrophoretic mobility shift assay of the isolated ACD of *Tli*VMA with two duplex DNAs containing the *Tli*VMA intein homing site. Purified ACD from *Tli*VMA intein was incubated at the indicated concentrations together with 5 µM of the 34-mer oligonucleotide duplex 1 (left) or oligonucleotide duplex 2 (right) for 2 h at 80 °C. M starnds for DNA size ladder.

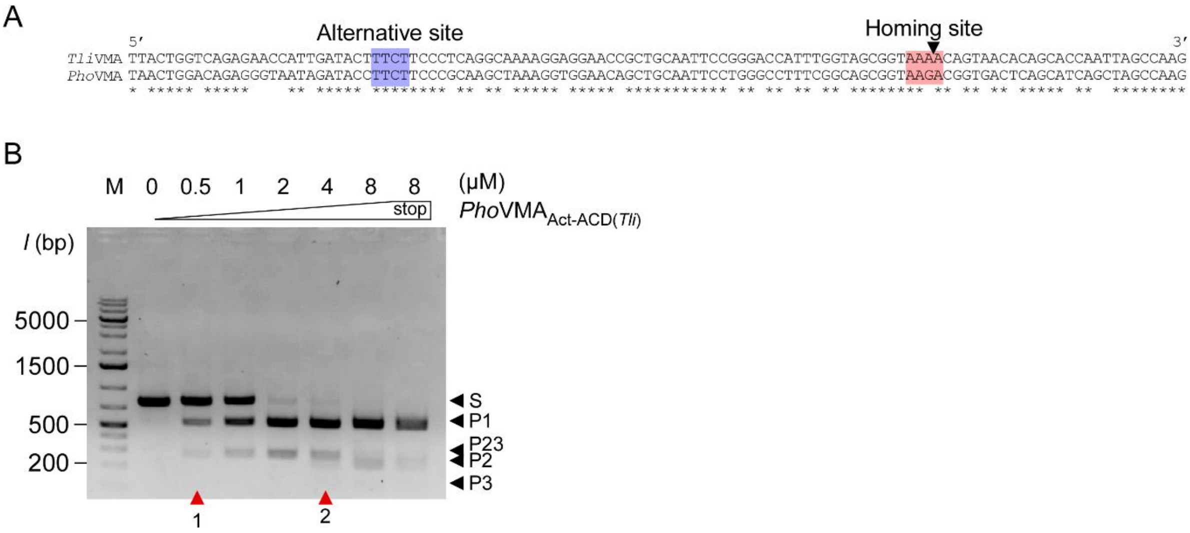

Supplementary Figure S6. Sequential processing of homing and alternative sites by *Pho*VMA$_{\text{Act-ACD(}Tli\text{)}}$ intein. (**A**) Sequence alignment of *Tli* and *Pho* VMA and substrate around homing (red) and alternative (blue) site. (**B**) Sequential processing of homing and alternative site in the 750-bp *Pho* DNA substrate by incubation with *Pho*VMA$_{\text{Act-ACD(}Tli\text{)}}$ intein at 80ºC for 2 h. S, substrate; P1, 500-bp product, P23, 250-bp intermediate, P2, ~200-bp product, P3, <75-bp product. Red arrowheads indicate the concentrations where cleavage of the homing site (1) and the alternative site (2) occurs.

Supplementary References

1. Kabsch W (2010) XDS. Acta Crystallogr Sect D Biol Crystallogr 66(2):125–132.
2. Panjikar S, Parthasarathy V, Lamzin VS, Weiss MS, Tucker PA (2005) Auto-Rickshaw : an automated crystal structure determination platform as an efficient tool for the validation of an X-ray diffraction experiment. Acta Crystallogr Sect D Biol Crystallogr 61(4):449–457.
3. Langer G, Cohen SX, Lamzin VS, Perrakis A (2008) Automated macromolecular model building for X-ray crystallography using ARP/wARP version 7. Nat Protoc 3(7):1171–9.
4. Arnold K, Bordoli L, Kopp J, Schwede T (2006) The SWISS-MODEL workspace: a web-based environment for protein structure homology modelling. Bioinformatics 22(2):195–201.
5. Emsley P, Lohkamp B, Scott WG, Cowtan K (2010) Features and development of Coot. Acta Crystallogr Sect D Biol Crystallogr 66(4):486–501.
6. Adams PD, et al. (2002) PHENIX : building new software for automated crystallographic structure determination. Acta Crystallogr Sect D Biol Crystallogr 58(11):1948–1954.
7. Svensson O, Malbet-Monaco S, Popov A, Nurizzo D, Bowler MW. (2015) Fully automatic characterization and data collection from crystals of biological macromolecules. Acta Crystallogr D Biol Crystallogr. 71(Pt 8):1757-1767.
8. McCoy AJ, Grosse-Kunstleve RW, Adams PD, Winn MD, Storoni LC, Read RJ. (2007) Phaser crystallographic software. J Appl Crystallogr. 40(Pt 4):658-674.
9. Karplus PA, Diederichs K. Linking crystallographic model and data quality. (2012) Science. 336(6084):1030-1033.
10. Diederichs K, Karplus PA. (1997) Improved R-factors for diffraction data analysis in macromolecular crystallography. Nat Struct Biol. 4(4):269-275.
11. Brünger AT. (1992) Free R value: a novel statistical quantity for assessing the accuracy of crystal structures. Nature. 355(6359):472-475.
12. Ellilä S, Jurvansuu JM, Iwaï H. (2011) Evaluation and comparison of protein splicing by exogenous inteins with foreign exteins in Escherichia coli. FEBS Lett. 585(21):3471-3477.
13. Muona M, Aranko AS, Iwai H. (2008) Segmental isotopic labelling of a multidomain protein by protein ligation by protein trans-splicing. Chembiochem. 9(18):2958-2961.
14. Holm L, Laakso LM. (2016) Dali server update. Nucleic Acids Res. 44(W1):W351-W355.
